# Dietary-Lifestyle Patterns Associated with Adiposity and Metabolic Abnormalities in Adult Men under 40 Years Old: A Cross-Sectional Study (MeDiSH Project)

**DOI:** 10.3390/nu12030751

**Published:** 2020-03-12

**Authors:** Marta Lonnie, Lidia Wadolowska, Elzbieta Bandurska-Stankiewicz

**Affiliations:** 1 Department of Human Nutrition, Faculty of Food Science, University of Warmia and Mazury in Olsztyn, 10-718 Olsztyn, Poland; lidia.wadolowska@uwm.edu.pl; 2 Department of Internal Medicine, Faculty of Medical Sciences, University of Warmia and Mazury in Olsztyn, 10-561 Olsztyn, Poland; bandurska.endo@gmail.com

**Keywords:** adiposity, adults, dietary patterns, lifestyle, men, metabolic, principle component analysis, young adults

## Abstract

The aim of this study was to examine the associations of dietary-lifestyle patterns (DLPs) with adiposity and metabolic abnormalities in adult Polish men that were under 40. The cross-sectional study included 358 men that were 19–40-year-old. Dietary and lifestyle data were collected with multicomponent food frequency questionnaire (KomPAN^®^). DPLs were derived with Principal Component Analysis (PCA) using 25 dietary and six lifestyle as the input variables. Adiposity was determined with the use of: overweight (body mass index 25–29.9 kg/m^2^), central obesity (waist-to-height ratio ≥ 0.5), general obesity (body fat ≥ 25%), excessive visceral fat tissue (≥ median), and increased skeletal muscle mass (≥ median). The metabolic abnormalities were characterised by elevated: fasting blood glucose (FBG ≥ 100 mg/dL), triglycerides (TG ≥ 150 mg/dL), total cholesterol (TC ≥ 200 mg/dL), or systolic or diastolic blood pressure (≥ 130 or ≥ 85 mmHg, respectively). Four PCA-driven DLPs were derived and labelled accordingly to the most characteristic dietary or lifestyle behaviours that were correlated with each pattern. Multivariate logistic regression revealed that higher adherence (upper vs. bottom tertile as referent) to “Protein food, fried-food, and recreational physical activity” pattern was associated with higher odds of overweight and increased skeletal muscle mass, and lower odds of: general obesity, excessive visceral fat tissue, and elevated TC. Higher adherence to “Healthy diet, active, past smokers” pattern was associated with higher odds of overweight and lower odds of: general obesity, excessive visceral fat tissue, and elevated FBG. Higher adherence to “Sandwiches and convenient diet” pattern was associated with higher odds of: central obesity, general obesity, excessive visceral fat tissue, elevated TC, elevated TG, occurrence at least two metabolic abnormalities, and lower odds of increased skeletal muscle mass. A higher adherence to “Fast foods and stimulants” pattern was associated with higher odds of central obesity, general obesity, excessive visceral fat tissue, and lower odds of increased skeletal muscle mass. The interrelations between diet and lifestyle behaviours were reflected in three out of four patterns. Healthy diet attempts combined with active lifestyle was associated with reduced risk of adiposity and metabolic abnormalities despite some unhealthy components, like former smoking or fried-food consumption. In contrary, patterns that were composed of undesirable dietary behaviours solely, as well as poor diet combined with stimulant use, were associated with higher adiposity and worse metabolic health, despite the relatively young age of the study participants. Accurate mapping of dietary-lifestyle behaviours can serve as a tool for formulating evidence-based recommendations.

## 1. Introduction

Young men are a population of a particularly increased risk of unfavourable health behaviours while going through the changes associated with the transition from adolescence to adulthood [1,2]. Leaving home, going to university, commencing employment and starting a family are milestones in life that can have detrimental health implications, contributing to the development of diet-related diseases, such as hypertension, obesity, diabetes, cardiovascular diseases, and cancers [3]. Moreover, it has been found that, in the young age group, men are at higher risk of fatal chronic conditions and heart diseases in comparison to women of the same age [4,5,6]. This phenomenon is believed to have not only behavioural background, but it is also associated with biological, hormonal, and genetic factors, which are sex-specific [7].

A Finnish study on men that were aged 18–29 years old revealed that, in early adulthood, unhealthy behaviours outweigh healthy and preferences for energy-rich foods are strong in this group, e.g., only small percentage of men consumed fruit and vegetables daily (13% and 8%, respectively), whereas a higher percentage consumed pizza and hamburgers more than once a week (24% and 19%, respectively) [8]. Starting university and living away from home has also been associated with low consumption of fruit and vegetables among male Polish students [9]. Moreover, while studying at university, male students have shown to engage more often than girls in addictive behaviours, such as excess alcohol drinking, smoking, and the use of stimulants, e.g., to enhance mental performance and cope with stress [10,11]. Poor coping strategies, which are reflected in addictive behaviours, can be also triggered by commencing first employment, which is often a source of stress for a young person. These results allow for hypothesizing, that young men are a specific population who engage in a mixture of explicit behaviours and, further, more detailed research is needed to fully understand the complex mechanisms of health-related decisions in this subpopulation.

Previous studies investigated the associations between predefined dietary and lifestyle risk factors and the occurrence of cardiovascular diseases, representing the a priori (hypothesis-driven) approach [12]. In brief, this approach is used to assess how closely selected behaviours match the widely accepted healthy lifestyle goals, e.g., has the recommendation for daily physical activity or fibre intake been met. One of the limitations of hypothesis-driven method is that it does not allow for picking out some unique combinations of behaviours, truly existing in studied populations. In contrast, an analysis of patterns provides the advantage of mirroring the real life scenario [13]. Furthermore, using the data-driven methods (the a posteriori approach) allows for revealing often-unexpected behavioural patterns that have previously not been hypothesised by the researchers. The a posteriori approach has been less frequently used and the interpretation of the results had some limitations. The majority of studies have combined both men and women to obtain overall patterns and were then investigating the differences in dietary patterns scores by gender [14]. However, as emphasised by Northstone and Emmett [15], a separate, gender-specific analysis of dietary patterns is highly recommended in European populations. In their study, the authors derived four dietary patterns in men: ‘Health conscious’, ‘Traditional’, Processed/confectionery’, and ‘Semi-vegetarian’. This study highlighted many interesting and important gender-specific differences in dietary patterns, e.g., ‘Traditional’ pattern was only evident in men, which indicated their strong preferences for traditional cooking, which were not present in women [15]. Unfortunately, the health outcomes of the adherence to these patterns or the associations with lifestyle factors have not been extensively explored.

Only a few studies attempted to investigate the associations between combined dietary-lifestyle patterns and metabolic abnormalities in young males [2,16,17]. However, the dietary component that was included in these studies was very limited, and comprised only few questions, usually regarding fruit and vegetable consumption (as an indicator of a healthy diet), with other important food groups being omitted. Nevertheless, the results from these studies provided valuable insight into clustering of dietary and lifestyle behaviours, suggesting that both healthy and unhealthy behaviours tend to cluster [14,15,16,17] and that the clustering of the least favourable to health behaviours was more prevalent in men, from younger age groups, and of a lower socioeconomic status [14].

To our knowledge, no previous studies included a broad range of dietary variables into the analysis of combined lifestyle and dietary behaviours, in the context of metabolic health and in the population of young men. The aim of this study was to examine the associations of dietary-lifestyle patterns (DLPs) with adiposity and metabolic abnormalities in young Polish men. The demographic that is described in this paper includes adult men up the age of 40. According to Levinson [18], this age marks the end of the early adulthood era and opens up the mid-life transition. For simplicity, we will refer to the group as young men throughout the paper.

## 2. Materials and Methods

### 2.1. Study Design and Participants

**Figure 1:**
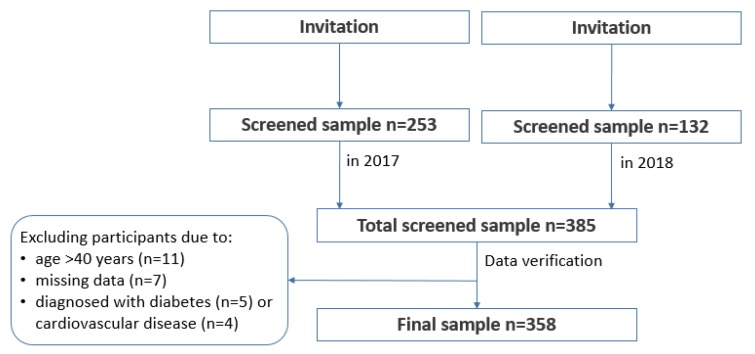
Study design and data collection.

The study was designed as a cross-sectional with convenience sampling. The participants were recruited through direct advertising, while using posters, leaflets, and communication with human resources departments of local employers. The recruitment was carried out in public and private sector workplaces that were located in Olsztyn city and around the city to cover urban, sub-urban, and rural areas of Warmia and Mazury region. The intent was to recruit men from different age groups, of all education levels and employment status (e.g., white vs. blue collars). The advertising of the study included sites, such as university campus, city council, unemployment office, health centres, retail, courier services, gas and electricity suppliers, etc., to achieve the maximum variability of the study sample in regards to demographic and socioeconomic characteristics and best reflect the structure of the general population. A proportion of recruited participants were enrolled through further referrals. The participants who took part in the study were recommending the study to their peers in person or through their social media channels. The inclusion criteria were: males, aged 19–40, with the cognitive ability to understand and respond to questions that were asked by the interviewer, written consent to participate. The exclusion criteria were: females, and a cognitive impairment that would not allow for understanding and responding to questions that were asked by the interviewer. A total sample screened consisted of 385 men. In the preliminary analysis, a total of 26 men were excluded from further analysis, due to missing data (*n* = 7), not meeting the age criterion on the day of screening (*n* = 11), reporting cardiometabolic diseases: cardiovascular disease (*n* = 4) and diabetes (*n* = 5). Although cardiometabolic diseases were not an exclusion criterion in the study, for the purpose of this paper it was decided that affected men should be removed from further analysis, due to small number of such subjects (9) and potential bias caused by the medication used. Figure 1 presents details of the sample collection.

All of the data regarding diet, lifestyle, and socioeconomic status were collected using closed-question from KomPAN^®^ questionnaire [19,20]. This multicomponent food frequency questionnaire was designed for a Polish population aged 15–65 years. KomPAN^®^ has been assessed in healthy and unhealthy subjects and confirmed to be a reliable tool with acceptable-to-very good reproducibility [20]. The data were collected in 2017 (January to March) and 2018 (April to May), during one-to one interviews with trained researchers.

### 2.2. Ethical Approval

The study was conducted within the Men’s Diet, Socioeconomic Status, and Health (MeDiSH) Project, which was approved by the Bioethics Committee of the Faculty of Medical Sciences, University of Warmia and Mazury in Olsztyn in December 8, 2016 (Resolution No. 45/2016) as an annex to ethical approval obtained in June 17, 2010 (Resolution No. 20/2010). Written informed consent to participate was obtained from all of the study participants.

### 2.3. Dietary and Lifestyle Behaviours

Data regarding the consumption frequency of 25 food groups were obtained. The respondents could choose one of six categories (next converted into daily frequency): never (0 times/day), 1–3 times a month (0.06 times/day), once a week (0.14 times/day), few times a week (0.5 times/day), once a day (1.0 time/day), or a few times a day (2.0 times/day), in accordance with questionnaire manual guide [19].

Lifestyle behaviours regarded daily frequency of meals, physical activity, smoking, and screen time. The respondents reported the number of daily eating occasions by choosing one from five answers, starting from one meal/day to five meals or more a day. The respondents could choose one of three categories to describe the level of physical activity at work or at school, as follows: (i) low—over 70% of time sedentary; (ii) moderate—about 50% of time sedentary and 50% active; and, (iii) higher—about 70% of time active or physical labour of high intensity. The respondents could choose one of three categories to describe the level of recreational physical activity, as follows: (i) low—mostly sedentary, watching TV, reading newspapers/book, light house works, walking for 1–2 h a week; (ii) moderate—walking, cycling, exercise, gardening, or other light intensity physical activity for 2–3 h a week; and, (iii) higher—cycling, running, gardening, or other sport activities that require physical activity for more than 3 h a week. Past and current smoking had a dichotomous choice of answers: yes or no. Screen time was assessed using question: ‘How many hours a day (on average) do you spend watching TV or using a computer (including work)?’ The respondents could choose one of six categories: < 2 h/day, 2 to < 4 h/day, 4 to < 6 h/day, 6 to < 8 h/day, 8 to < 10 h/day, and ≥ 10 h/day. For each answer of each lifestyle variable, separately, numerical values were assigned, as follows: 1, 2, etc., to used them in statistical analysis.

### 2.4. Adiposity and Metabolic Assessment

Trained researchers undertook the measurements of body weight, size, and according to the International Society for Advancement of Kinanthropometry (ISAK) International Standards for Anthropometric Assessment guidelines [21]. A professional equipment and measuring tape were used: for measuring height—a portable stadiometer SECA 220, for weight—electronic digital scale SECA 799, for waist circumference—stretch-resistant tape SECA 201 to measure on bare sin, just above the iliac crest, for body fat and skeletal muscle mass (using bioelectrical impedance technique)—body composition analyser SECA medical Body Composition Analyze (mBCA) 515. Adiposity was determined based on several commonly used anthropometric indices: overweight (body mass index, BMI = 25–29.9 kg/m^2^), central obesity (waist-to-height ratio, WHtR ≥ 0.5), and general obesity (body fat ≥ 25%) [22,23,24]. The median values (Me) were applied to assess excessive visceral fat tissue (≥ Me of fat tissue volume, i.e., 1.565 l) and increased skeletal muscle mass (≥ Me of body mass, i.e., 37%).

Measuring the concentration of fasting blood glucose (FBG), triglycerides (TG), and total cholesterol (TC) in capillary blood was undertaken to collect the metabolic outcomes. The tests were performed by piercing the fingertip to draw blood, applying the blood to a chemically active disposable test strip, and then placing it into an Accutrend Plus glucometer (Roche Diagnostic GmbH, Mannheim, Germany). All of the tests were performed in the morning and the participants were asked to restrain from eating or drinking (apart from water) up to 8–10 h before the appointment. The measurements of systolic (SBP) and diastolic blood pressure (DBP) were taken. It was based on repeated measures using electronic monitor (Omron M3 Intellisense Automatic Blood Monitor, Omron Healthcare, Mannheim, Germany), according to the National Institute for Health and Care Excellence (NICE) procedures [25], meaning that all of the measurements were being obtained in a uniform manner for each participant to minimise the bias. Metabolic abnormalities were recorded if the following markers were elevated: FBG ≥ 100 mg/dL, TG ≥ 150 mg/dL, TC ≥ 200 mg/dL, or systolic or diastolic blood pressure ≥ 130 or ≥ 85 mmHg, respectively [26,27,28].

### 2.5. Confounding Variables

Literature on dietary patterns and health was reviewed to identify relevant confounders to prevent the distortion of the true relationship between exposures and outcomes and minimize potential bias. The considered confounding variables, which were used in further analysis, were age as continuous variable and categorical socioeconomic variables: place of residence (four categories: village, town < 20,000, town 20,000 to 100,000, city > 100.000 inhabitants), education (three categories: lower secondary, upper secondary, higher), and financial situation (four categories). Financial situation was assessed while using the following question: ‘How would you describe your household’s overall situation?’ Five answers were given to choose one from: (i) ‘we do not have enough money for basic needs’—nobody reported such situation; (ii) ‘we have to be very careful with our daily budget’; (iii) ‘we have enough money for our daily needs, but we need to budget for bigger purchases’; (iv) ‘we have enough money for our needs without particular budgeting’; and, (v) ‘we can afford some luxury’.

### 2.6. Statistical Analysis

Sample size calculation was based on the occurrence of adiposity and metabolic abnormalities. A database that was collected in 2017 and covering 253 participants was used. Assuming a two-sided significance level of 0.05 and 80% power to detect a 50% difference in the occurrence of adiposity and metabolic abnormalities between two groups (moderate or higher adherence to the DLP vs. lower adherence as reference), the sample size was, for example: 75 for overweight, 97 for elevated BP, 132 for elevated TC, 146 for general obesity, and 165 for elevated TG, per each group. Thus, taking the use of multiple markers to assess adiposity and metabolic abnormalities into account, we have found that the sample size was sufficient for detecting differences between groups, if they exist, except for elevated FBG, which should be interpreted with caution.

Data were presented as percentages of the sample for categorical variables and the means and standard deviations (SDs) for continuous variables with normal distribution (e.g., age, adiposity, and metabolic markers) or medians and interquartile ranges (IQRs) for continuous variables without normal distribution (e.g., food frequency consumption expressed in times/day). Before statistical analysis, variables normality was verified with two tests: Kolmogorov-Smirnov test and Shapiro-Wilk test. Differences between groups were verified with Pearson’s chi-squared for categorical variables, T test for continuous variables with normal distribution or Kruskal-Wallis test for continuous variables without normal distribution.

The DLPs were derived using Principal Component Analysis (PCA), with varimax normalized rotation [29]. A total of 31 variables were included in the PCA: 25 dietary- and six lifestyle-related. During the identification of the number of DLPs, the following criteria were considered: (i) the eigenvalues of the variable correlations >1.0, (ii) the plot of eigenvalues, and (iii) the total variance explained [29]. Rotated factor loadings with an absolute value ≥ |0.30| were considered to be specific to the given pattern and used to label the patterns accordingly. The higher values of factor loadings, the stronger association between dietary or lifestyle variables and the DLP. For each subject and each pattern, DLP scores were calculated as a product of factor loading and food frequency consumption (for dietary variables) or numerical categories assigned (for lifestyle variables). Next, for each DLP, tertile intervals were calculated which aimed to categorise subjects’ adherence to the patterns: subjects that were located in the upper tertile were characterised as those with higher adherence to the pattern, while subjects located in the bottom tertile as those with lower adherence. The percentage distribution of adiposity and metabolic abnormalities was analysed across the tertiles of DLPs while using Pearson’s chi-squared test.

Logistic regression verified the associations between DLPs and adiposity or metabolic outcomes. The odds ratios (ORs) and 95% confidence intervals (95%CIs) were calculated. Two models were created: crude and adjusted for potential confounders: age, place of residence, financial situation, and education (see Section 2.4). The modelled variables (see Section 2.3) were:

(1) related to adiposity: overweight (reference (ref.): normal weight), central obesity (ref.: without), general obesity (ref.: without), excessive visceral fat tissue ≥ median of fat tissue volume (ref.: < Me), and increased skeletal muscle mass ≥ median of body mass percentage (ref.: < Me), and
(2) related to metabolic abnormalities: elevated FBG (ref.: not elevated), elevated TG (ref.: not elevated), elevated TC (ref.: not elevated), elevated SBP or DBP (ref.: both not elevated), at least two metabolic abnormalities (ref.: no metabolic abnormalities).

With respect to the subjects’ adherence to the DLPs, the modelled categories were moderate or higher adherence while reference category (OR = 1.00) was lower adherence (i.e., the bottom tertile). The statistical analysis was performed while using STATISTICA software (version 10.0 PL; StatSoft Inc., Tulsa, USA; Kraków, StatSoft Polska). A *p*-value < 0.05 was considered to be statistically significant.

## 3. Results

### 3.1. Sample Characteristics

The consumption of at least four meals a day was reported by approx. 67% of the sample, high level of physical activity at work or at school by approx. 18%, high level of recreational physical activity by approx. 41%, and screen time lasting ≥ 8 h/day by 28.5% (Table 1). Over 15% of men reported consumption of foods with frequency a few times a day for: butter, refined bread, vegetables, milk, and fruit, and over 40% of men never consumed: lard, energy drinks, and tinned meats (Table 2). Means and medians (with interquartile ranges) of foods frequency consumption (times/day) can be found in supplementary material (Table S1).

Overweight was found in approx. 45% of men, central obesity in approx. 40%, while general obesity measured with body fat content in approx. 32% (Table 3). Among the metabolic outcomes, the most common abnormality was elevated TC (approx. 34%), followed by elevated TG (approx. 30%), and FBG (approx. 11%). Elevated diastolic or systolic blood pressure was found in almost 40% of the sample, and two or more metabolic abnormalities in just over 20% of the study sample.

### 3.2. Dietary-Lifestyle Patterns

**Figure 2:**
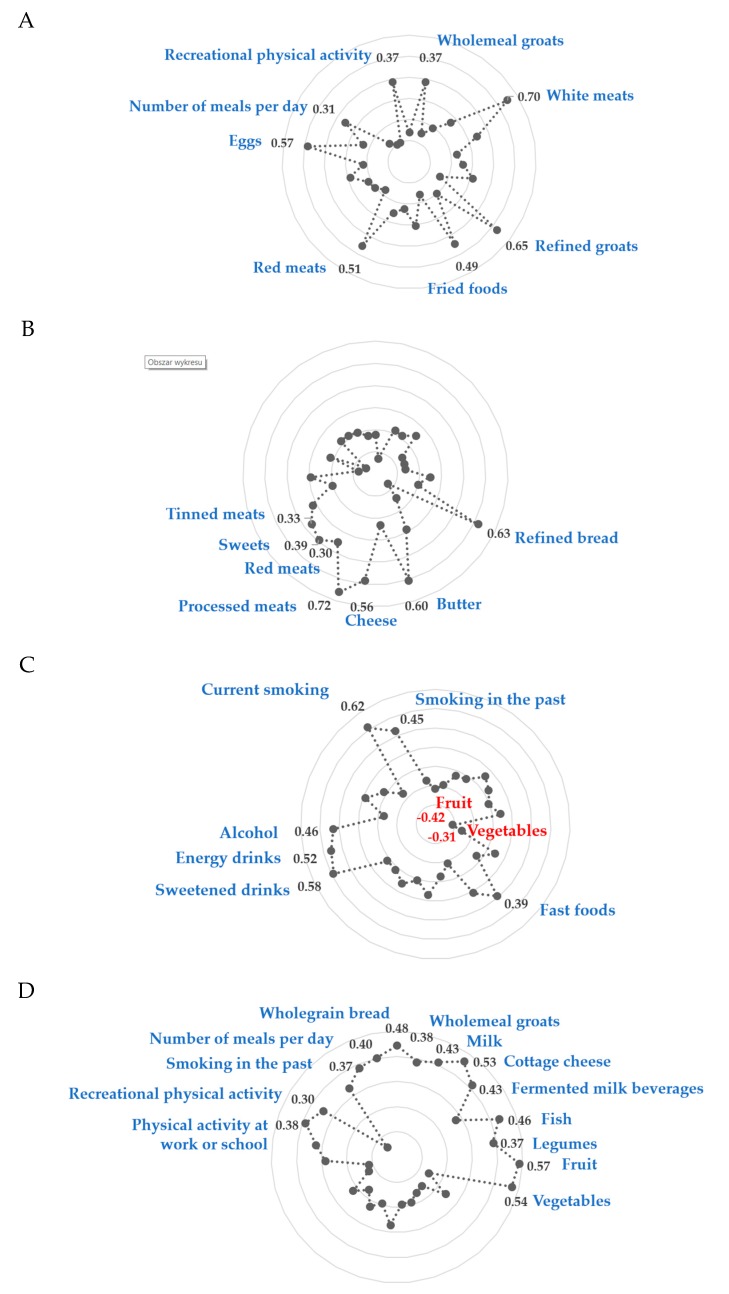
Diagrams of factor loadings that characterise each dietary-lifestyle pattern identified with principal component analysis. (**A**)—“Protein food, fried-food and recreational physical activity” pattern; (**B**)—““Sandwiches and convenient diet” pattern; (**C**)—““Fast foods and stimulants” pattern; (**D**)—““Healthy diet, active, past smokers” pattern; Only factor loadings of >|0.30| are shown for simplicity. Total variance explained by four dietary-lifestyle patterns is 32.2%. The factor loadings for “lard” and “screen time” were <|0.30| in all factors, hence the data are not shown.

Four dietary-lifestyle patterns were derived with a total variance explained of 32.2% (Figure 2, Table 4; Supplementary Material: Table S2). The “Protein food, fried-food, and recreational physical activity” pattern was characterized by the frequent consumption of white meats (factor loading = 0.70), refined groats (0.65), eggs (0.57), red meats (0.51), fried foods (0.49), wholemeal groats and (0.37), as well as more frequent meals throughout the day (0.37) and a higher level of recreational physical activity (0.31). The “Sandwiches and convenient diet” pattern was characterized by the frequent consumption of processed meats (0.72), white bread (0.63), butter (0.60), cheese (0.56), sweets (0.39), tinned meats (0.33), and red meats (0.30). The “Fast foods and stimulants” pattern was characterized by frequent consumption of sweetened beverages (0.58), energy drinks (0.52), alcohol (0.46), fast foods (0.39), and less frequent consumption of fruit (−0.42) and vegetables (−0.31). Men from this pattern were also described as current (0.62) and/or former (0.45) smokers. The “Healthy diet, active, past smokers” pattern was characterized by the frequent consumption of fruit (0.57), vegetables (0.54), fermented milk beverages (0.53), wholegrain bread (0.48), fish (0.46), cottage cheese (0.43), milk (0.42), wholegrain groats (0.38), and legumes (0.37), as well as more frequent meals throughout the day (0.40), former smoking (0.37), and higher level of physical activity at work or school (0.38) and in the recreational time (0.30). 

The Supplementary Material (Tables S3–S6) present the medians (interquartile range) and sample distribution (%) of DLP components by adherence to each dietary-lifestyle pattern.

### 3.3. Associations between DLPs and Adiposity and Metabolic Outcomes

In the adjusted model, higher subjects’ adherence (upper vs. bottom tertile as reference) to the “Protein food, fried-food and recreational physical activity” pattern was associated with higher odds of overweight (OR 2.22, 95% CI: 1.19–4.15) and increased skeletal muscle mass (2.02, 1.17–3.50), and lower odds of: general obesity (0.23, 0.11–0.45), excessive visceral fat tissue (0.45, 0.26–0.79), and elevated TC (0.44, 0.25–0.79). Higher subjects’ adherence to “Healthy diet, active, past smokers” pattern was associated with higher odds of overweight (3.35, 1.82–6.18) and lower odds of: general obesity (0.38, 0.19–0.74), excessive visceral fat tissue (0.51, 0.29–0.89), and elevated FBG (0.32, 0.11–0.92). Higher subjects’ adherence to the “Sandwiches and convenient diet” pattern was associated with higher odds of: central obesity (1.99, 1.14–3.47), general obesity (3.45, 1.77–6.83), excessive visceral fat tissue (2.59, 1.48–4.54), elevated TC (2.72,1.53–4.86), elevated TG (1.87, 1.03–3.39), occurrence at least two metabolic abnormalities 2.54 (1.20-5.39), and lower odds of increased skeletal muscle mass (0.53, 0.31–0.90). Higher subjects’ adherence to the “Fast foods and stimulants” pattern was associated with higher odds of central obesity (2.07, 1.13–3.78), general obesity (4.76, 2.10–10.74), excessive visceral fat tissue (3.17, 1.65–5.98), and lower odds of increased skeletal muscle mass (0.48, 0.27–0.86) (Table 5 and Table 6).

The Supplementary Material presents the occurrence of adiposity and metabolic abnormalities (%) by adherence to each dietary-lifestyle pattern as well as crude associations (unadjusted models) between dietary-lifestyle patterns (DLPs) and adiposity and metabolic abnormalities (Tables S7–S9).

## 4. Discussion

Among the derived patterns, only the “Fast foods and stimulants” pattern was consistent in terms of combining unhealthy dietary and lifestyle behaviours. Two patterns were composed of mixed behaviours, e.g., healthy diet with some unhealthy lifestyle behaviours from the past (“Healthy diet, active at work, past smokers”) or healthy lifestyle with only a relatively healthy diet (“Protein food, fried-food, and recreational physical activity”). The “Sandwiches and convenient diet” pattern did not include any of the lifestyle components. The results of this study revealed that dietary-lifestyle patterns are associated with adiposity and metabolic health outcomes in young men. An increased risk of obesity was observed among men with high adherence to the “Fast foods and stimulant” and the “Sandwiches and convenient diet” patterns, with the latter also being associated with the occurrence of metabolic abnormalities. “Protein food, fried-food and recreational physical activity” and “Healthy diet, active, past smokers” patterns were associated with a reduced risk of obesity and metabolic abnormalities.

The use of a data-driven approach facilitated the identification of new, unique patterns of dietary and lifestyle behaviours in young men. Surprisingly, in this sex- and age-specific sample, dietary behaviours were not entirely comparable with dietary patterns that were previously described in the literature (such as “Western” or “Traditional” pattern) [30,31]. Only the dietary components of “Healthy diet, active at work, past smokers” DLP were consistent with the composition of a dietary pattern commonly labelled as “Prudent” [32,33]. Nevertheless, despite some similarities, the comparison to previous reports is limited, since the patterns derived in the current study were also composed of lifestyle components—absent in the traditional approach of studying dietary patterns.

The “Protein food, fried-food, and recreational physical activity” pattern that was composed of predominantly dietary sources of protein was complemented by a high level of recreational physical activity and consuming a higher number of meals a day. This specific pattern of dietary-lifestyle behaviours was associated with the most desirable body composition parameters. The men from this pattern were more likely to be overweight according to BMI, however extra body weight was not a result of excessive body fat content (odds ratio: 0.23) or central obesity (by WC: 0.39), but it was attributed to the higher skeletal muscle mass (2.02). The components of this pattern suggest that men from this group complemented their recreational physical activity with a diet high in protein, presumably to enhance the muscle protein synthesis during exercising. The adherence to this dietary pattern was also associated with reduced risk of elevated total cholesterol (by 54%), which suggested the potential health-promoting effect on the blood lipid profile. Interestingly, this pattern was also comprised of less-desirable components, such as fried-foods, indicating that there were areas worthwhile addressing, perhaps while formulating the recommendation for this group, e.g., opting for grilled meat or partially replacing it with plant-based proteins, found in legumes. Furthermore, including more fruit and vegetables in the diet could contribute to reducing the oxidative stress that is associated with exercise [34]. Nevertheless, the observations within this pattern allow for a hypothesis that physical activity might mitigate the effects of some dietary behaviours that are not in line with dietary guidelines.

This observation was somewhat confirmed when analysing the associations between “Healthy diet, active, past smokers” and adiposity-metabolic outcomes. The dietary components of this pattern most closely matched the profile of dietary guidelines, and they were complemented by a higher level of occupational and recreational physical activity, and regular meals. Interestingly, men with the highest adherence to this pattern were also characterised as former smokers, which indicated that a behavioural change had occurred at some point in their lives, directing them towards a healthier lifestyle. The potentially alleviating effect of physical activity combined with healthy diet was observed in this pattern and it was reflected in the lower odds of central obesity (by WC: 0.33), excessive body fat content (0.38) and lower volume of visceral fat tissue (0.51), as well as lower risk of elevated blood glucose level (0.32). Similarly to the previously described pattern, a higher risk of being overweight (3.35) was observed in this group, which suggested that the sole use of BMI is not sufficient for identifying adiposity-related risks.

The two patterns that were associated with negative health outcomes were: “Sandwiches and convenient diet” and “Fast food and stimulants”. Men with higher adherence to the “Sandwiches and convenient diet” pattern had higher odds of central obesity (by WC: 2.78), excessive body fat content (3.45), an excessive volume of visceral fat (2.59), accompanied by lower skeletal muscle mass (0.53). Interestingly, the high adherence to this pattern was not reflected in the adiposity risks that were assessed with BMI. This is of particular concern, since it is the most commonly used assessment in clinical practice [35]. In this group of men, muscle mass was compensated with adipose tissue (most likely centrally accumulated), which resulted in the absence of clear external clues; in the literature, this phenotype is often described as TOFI (thin-on-the-outside fat-on-the-inside) [36]. Therefore, the self-perceived obesity risk or health risks that were assessed in the medical setting could have been masked, and not addressed with additional screening. As expected, men with a high adherence to this pattern had the least favourable metabolic profile, with a high risk of elevated total cholesterol (2.72) and triglycerides (1.87) concentrations. This was the only pattern without the lifestyle components, which suggested that this group might not have been homogenous enough to reveal any correlations between lifestyle and dietary behaviours. However, when the percentage distribution of study sample across the tertiles of this DLPs was analysed (Supplementary Material: Table S4), it can be observed that these men were characterised by a low level of physical activity at work and in their free time. It could be hypothesized that men from this pattern had a sedentary type of job and displayed low interest (or lack of time) in physical activity after work. Inactive behaviour was complemented with a convenient diet, which was composed of bread, butter, cheese, processed meats, and sweets, which suggested low diet variety and snacking throughout the day.

A pattern with the most undesirable dietary-lifestyle composition was characterised by the frequent intake of sweetened beverages, energy drinks, alcohol, and fast foods, along with current and past smoking, hence it was labelled “Fast foods and stimulants’. Surprisingly, the associations with this pattern were only significant in terms of adiposity, but not with metabolic outcomes. Men with higher adherence to this pattern were more likely to be obese (by BMI: 3.17; WC: 3.60), had high body fat content (4.76) and excessive visceral fat tissue (3.17), and lower odds of higher muscle mass (0.48). The clustering of unhealthy dietary behaviours is not uncommon. Previous studies concluded, that, particularly in men, unhealthy diet clusters with substance use, which might have psychological, social or biological background [37]. Perhaps, the age of men from this pattern played a role in postponing health-related consequences.

### 4.1. Strengths and Limitations

To our knowledge, this is the first study to examine the associations between a broad range of dietary (25 variables) and lifestyle (6) behaviours and metabolic health in the population of adult men under 40. The study used multiple measures to assess adiposity status (including the assessment of body composition using bioelectrical impedance technique with body composition analyser SECA mBCA 515), thus confirming that a sole use of BMI might not detect early risks, especially if a higher body mass results from a higher content of muscle mass.

Despite the moderate number of subjects (> 350), the sample size met the criteria for performing multidimensional statistical analysis that requires ‘big data’, i.e., the recommended 10:1 subject-to-item ratio for principal component analysis; in our study the ratio was 11.5:1, i.e., 358 of subjects, 31 of input variables [38]. The sample size was also sufficient for regression analysis, including the ‘one in ten rule’, for all of the independent variables. Furthermore, minimal sample size was calculated before conducting the study and, next, its adequacy was checked for data covering all study participants (n = 358). The post-hoc statistical power was calculated. For example, when compared the occurrence of overweight (39.8% vs. 55.4%), central obesity (52.2% vs. 35.5%), excess of visceral fat tissue (64.4% vs. 39.7%), elevated TG (38.1% vs. 25.6%), or at least two metabolic abnormalities for extreme groups of adherence, assuming a 5% significance level, the statistical power was 68%, 74%, 97%, 55%, or 86%, respectively. In general, we have found that the sample size (*n* = 358) was sufficient for detecting differences between groups, if they exist, with one exception. The occurrence of elevated FBG requires a larger sample to detect the differences between groups, if they exist (for our data power 10% to 38%), so these data should be interpreted with caution.

The main limitation of our study is the use of the descriptive qualitative food frequency questionnaire (FFQ). This type of questionnaire does not provide information on the estimated portion sizes, but it is widely used in studying dietary patterns, particularly in settings with limited resources [39]. In Poland, there is only one validated semi-quantitative FFQ [40], however the process of data collection with the use of this questionnaire is estimated to last between 3 to 4 h, which, from our past experience, was discouraging to the respondents, compromising the quality of the reported data. This, combined with extra time spent on data collection regarding socioeconomic status, medical history, as well as blood tests and anthropometric measures, would result in a lower participation level. Hence, we used a validated descriptive qualitative FFQ to minimise respondents’ burden and increase the level of participation in the study, which, despite its limitations, is a quicker, inexpensive method, previously shown to be correlated with data that were obtained with semi-quantitative FFQs [41,42,43,44].

Secondly, our estimations would be more precise and have higher external validity if a larger, representative sample was recruited. It is uncertain how closely the recruited sample represents the general population of Polish men 19-40-years-old, since it was not possible to carry out comparative statistical analysis for demographic and socioeconomic variables (education, financial situation, residence), due to the lack of national data for this specific subsample. The same applies to the occurrence of adiposity and metabolic abnormalities. Nevertheless, the association of the specific patterns with adiposity and metabolic abnormalities can be still analysed and its potential physiological mechanisms may be further investigated.

Finally, it would be interesting to investigate the associations of the derived dietary-lifestyle patterns with more advanced markers of metabolic health, such as detailed blood lipid profile (high-density lipoproteins-HDL and low-density lipoproteins-LDL), hemoglobin A1c, and insulin levels, as well as inflammation markers (e.g., interleukin-6, C-reactive protein) [45,46]. However, the main aim of the current study was to identify dietary-lifestyle patterns in the population of men <40 and verify the associations with metabolic health, as measured with the simple, inexpensive tests, listed as signposts of metabolic syndrome and commonly used in general medical practice. We hope that our results provide a good basis for other researchers to design further investigations that combine dietary-lifestyle patterns with more advanced biochemical markers.

### 4.2. Practical Implications

We believe that the results of our study have practical implications. Analysing the complex matrix of lifestyle behaviours reveals its multicomponent and interconnected nature, which is not always apparent. There is a possibility that the traditional approach in developing healthy lifestyle messages might not meet the needs of certain groups of men. A better understanding of these matrices of dietary and lifestyle behaviours in the targeted population might result in developing more effective interventions, by correcting specific behaviours rather than the entire lifestyle.

Although the results of our study are an important literature contribution for both parties, public health nutritionists, and clinicians, the findings do not have direct application in clinical practice. The accurate mapping of dietary-lifestyle behaviours can serve as a tool for formulating evidence-based recommendations, hence its application is more suitable for preventative care and health education purposes. The main goal of our analysis was to investigate which combination of behaviours stipulate the highest risk of metabolic abnormalities. Having this knowledge will help to predict, for example, that men <40 years old who smoke cigarettes, are very likely to drink energy drinks and eat fast-foods. Hence, instead of providing standard dietary recommendations (e.g., increasing fruit and vegetable intake etc.), perhaps more focus should be first placed on identifying how to tackle stimulant use, and then the second step could include a gradual introduction of traditional dietary and lifestyle recommendations. However, knowledge regarding which messages are effective for this group would have to be evaluated in a separate study. The current paper only provides a population characteristics and theoretical framework.

## 5. Conclusions

The novelty of our study lies in the identification of unique sets of coexisting dietary and lifestyle behaviours in the population of men under 40 years old. Furthermore, these previously unmapped mixtures of behaviours were associated with adiposity and metabolic health outcomes. In the study sample, the interrelations between diet and lifestyle behaviours were reflected in three out of four patterns. Interestingly, none of the patterns could be recognised as being unequivocally healthy. Our findings strengthen previous evidence showing the association of healthy diet and active lifestyle with a declined occurrence of adiposity and metabolic abnormalities. An unexpected result is that healthy diet attempts combined with active lifestyle, at work or leisure time, were associated with a reduced risk of adiposity and metabolic abnormalities, despite some unhealthy components, like former smoking (“Healthy diet, active at work, past smokers”) or fried-food consumption (“Protein food, fried-food and recreational physical activity”). On the contrary, patterns that were composed of undesirable dietary behaviours solely (“Sandwiches and convenient diet”), as well as poor diet combined with stimulant use (“Fast foods and stimulants”), were associated with higher adiposity and worse metabolic health, despite the relatively young age of the study participants.

Similarly to research investigating the synergies between singular nutrients found in foods [47], there is a need for exploring the synergies between diet and other lifestyle behaviours, and the associations of these complex matrices with adiposity and metabolic health. Thus, our results give rise to the design of further investigation by other researchers. Further studies could investigate a wider range of metabolic health markers as an outcome of dietary-lifestyle patterns.

## Figures and Tables

**Table 1:** Sample characteristic by sociodemographic and lifestyle variables and age groups.

Variables	Percentage of the Sample (%) Age Groups [Years]			*p*-Value
	Total	19–30 Years	31–40 Years	
Number of subjects	358	176	182	
**Sociodemographic variables**				
**Place of residence**				****
Village	20.4	26.1	14.8	
Town (<100,000)	15.9	23.3	8.8	
Big city	63.7	50.6	76.4	
**Education**				****
Secondary or lower	41.9	58.0	26.4	
Higher	58.1	42.0	73.6	
**Financial situation ^1^**				ns
Modest	27.1	26.2	28.0	
Comfortably	64.2	61.9	66.5	
Wealthy	8.7	11.9	5.5	
**Lifestyles behaviours**				
**Number of meals per day**				ns
1–2	4.4	3.9	3.8	
3	28.5	29.5	29.7	
4	42.2	39.2	39.0	
5 or more	24.9	27.4	27.5	
**Physical activity at work or at school ^2^**				ns
Low	50.0	47.7	52.2	
Moderate	31.8	33.0	30.8	
High	18.2	19.3	17.0	
**Recreational physical activity ^3^**				**
Low	15.4	13.1	17.6	
Moderate	43.8	36.9	50.5	
High	40.8	50.0	31.9	
**Current smoking**	15.9	18.2	13.7	ns
**Smoking in the past**	38.5	35.2	41.8	ns
**Screen time** (hours/day) **^4^**				****
<2	10.9	8.0	13.7	
2 to <4	20.7	26.7	14.8	
4 to <6	24.0	30.7	17.6	
6 to <8	15.9	16.5	15.4	
8 to <10	17.6	10.8	24.2	
≥10	10.9	7.4	14.3	

**^1^** Financial situation was assessed using the question: ‘How would you describe your household’s overall situation?’; The ‘modest’ category consisted of two answers: ‘we have to be very careful with our daily budget’ and ‘we have enough money for our daily needs, but we need to budget for bigger purchases’; The ‘comfortably’ category consisted of one answer: ‘we have enough money for our needs without particular budgeting’; The ‘wealthy’ category consisted of one answer: ‘we can afford some luxury’. **^2^** Physical activity at work or at school was categorised as follows: low—over 70% of time sedentary; moderate—about 50% of time sedentary and 50% active; higher—about 70% of time active or physical labour of high intensity; **^3^** Recreational physical activity was categorised as follows: low – mostly sedentary, watching TV, reading newspapers/book, light house works, walking for 1–2 h a week; moderate—walking, cycling, exercise, gardening, or other light intensity physical activity for 2–3 h a week; higher—cycling, running, gardening, or other sport activities that require physical activity for more than 3 h a week; **^4^** Screen time was assessed using the question: ‘How many hours a day (on average) do you spend watching TV or using a computer (including work)?’ Statistical significance (Person’s chi-squared test): ** *p* < 0.01, **** *p* < 0.0001; ns—statistically insignificant.

**Table 2:** Sample characteristic by dietary behaviours (*n* = 358).

Foods ^1^	Frequency Consumption (% of the Sample)					
	Never	1–3 Times a Month	Once a Week	Few Times a Week	Once a Day	Few Times a Day
Butter	12.3	10.1	6.1	18.7	29.9	22.9
Refined bread	4.5	14.2	14.5	25.4	22.6	18.7
Vegetables	0.8	2.2	7.5	34.9	35.8	18.7
Milk	7.3	10.6	12.0	28.5	25.1	16.5
Fruit	0.0	3.9	12.6	33.2	35.2	15.1
Processed meats	3.1	3.1	12.0	41.3	29.6	10.9
Wholemeal bread	5.3	16.8	17.3	32.1	18.7	9.8
Refined groats	2.0	13.4	27.4	40.5	11.2	5.6
Sweets	2.5	12.8	24.9	32.4	22.9	4.5
Eggs	1.7	8.9	24.0	45.3	15.4	4.7
Fermented milk beverages	6.7	21.5	17.9	32.1	17.6	4.2
Sweetened drinks	12.6	38.3	20.1	17.3	7.8	3.9
White meats	1.4	4.5	13.7	62.8	14.0	3.6
Cheese	3.4	9.5	19.8	47.8	16.8	2.8
Cottage cheese	7.8	20.7	27.7	33.5	7.5	2.8
Fried foods	1.4	7.5	19.3	52.8	16.8	2.2
Wholemeal groats	7.0	32.7	21.5	27.1	9.8	2.0
Red meats	3.1	22.1	23.5	41.3	8.9	1.1
Energy drinks	50.8	34.1	6.7	7.3	0.6	0.6
Fish	3.4	37.2	41.3	15.9	1.7	0.6
Alcohol	5.3	35.8	37.7	19.3	1.7	0.3
Fast foods	9.5	64.2	18.7	7.0	0.3	0.3
Lard	62.0	26.3	6.7	4.5	0.3	0.3
Legumes	10.3	56.1	23.2	8.4	1.7	0.3
Tinned meats	41.3	45.8	9,5	2.2	1.1	0.0

^1^ Foods sorted by sample percentages of ‘few times a day’ category.

**Table 3:** Sample characteristic by the occurrence of adiposity and metabolic abnormalities and age groups: means (SD) and sample percentage distribution (%).

Variables	Total	Age Groups		*p*-Value
		19–30 Years	31–40 Years	
Number of subjects	358	176	182	
Age (years): mean (SD)	30.1 (5.9)	24.8 (3.2)	35.2 (2.5)	****
**Adiposity outcomes: mean (SD)**				
BMI (kg/m^2^)	26.0 (3.7)	25.3 (3.8)	26.6 (3.4)	***
WC (cm)	89.9 (10.4)	87.4 (10.4)	92.4 (9.9)	****
WHtR (-)	0.50 (0.06)	0.48 (0.06)	0.51 (0.06)	****
Body fat (%)	22.2 (6.8)	20.5 (7.1)	23.9 (6.1)	****
Visceral fat tissue (l)	1.96 (2.21)	1.60 (1.88)	2.30 (2.44)	**
Skeletal muscle mass (%)	36.8 (4.0)	37.8 (4.2)	35.9 (3.5)	****
**Adiposity outcomes** **: percentage of the sample (%)**				
Overweight (BMI = 25-29.9 kg/m^2^)	45.5	37.5	53.3	**
Central obesity (WHtR ≥ 0.5)	40.5	26.1	54.4	****
General obesity (Body fat ≥ 25%)	32.4	23.3	41.2	****
Excess of visceral fat tissue (≥ Me, i.e., 1.565 l)	50.6	36.4	64.3	****
Increased skeletal muscle mass (≥ Me, i.e., 37%)	50.0	61.9	38.5	****
**Metabolic outcomes: mean (SD)**				
FBG (mg/dL)	85.0 (13.4)	84.1 (12.6)	85.9 (14.1)	ns
TG (mg/dL)	143.1 (99.3)	126.7 (77.7)	159.0 (114.5)	**
TC (mg/dL)	185.6 (40.2)	175.2 (40.4)	195.7 (37.5)	****
SBP (mmHg)	126.1 (12.0)	125.1 (11.9)	127.1 (12.1)	ns
DBP (mmHg)	77.4 (9.5)	74.1 (9.0)	80.6 (8.9)	****
**Metabolic outcomes: percentage of the sample (%)**				
Elevated FBG (≥ 100 mg/dL)	10.6	8.5	12.6	ns
Elevated TG (≥ 150 mg/dL)	29.6	24.4	34.6	ns
Elevated TC (≥ 200 mg/dL)	34.1	23.3	44.5	***
Elevated SBP (≥ 130 mmHg) or DBP (≥ 85 mmHg)	39.9	35.2	44.5	
No metabolic abnormalities	27.9	37.5	18.7	***
1 metabolic abnormality	41.3	42.0	40.7	ns
2 metabolic abnormalities	20.7	12.5	28.6	****
3 metabolic abnormalities	8.7	7.4	9.9	ns
All metabolic abnormalities	1.4	0.6	2.2	ns

BMI—body mass index; WC—waist circumference, WHtR—waist to height ratio; DBP—diastolic blood pressure; FBG—fasting blood glucose; TG—triglycerides; TC—total cholesterol; SBP—systolic blood pressure; SD—standard deviation. Statistical significance (T test or Person’s chi-squared test): ** *p* < 0.01, *** *p* < 0.001, **** *p* < 0.0001; ns—statistically insignificant.

**Table 4:** Distribution of dietary and lifestyle components of dietary-lifestyle patterns (DLPs) across higher adherence to the DLPs (% of the sample).

Components ^1^ of DLPs	Higher Adherence to the DLPs				Significance of the Relation between the DLPs
	Protein Food, Fried-Food and Recreational Physical Activity (A)	Sandwiches and Convenient Diet (B)	Fast Foods and Stimulants (C)	Healthy Diet, Active, Past Smokers (D)	
Number of the subjects	121	121	121	121	
**Frequency consumption of:**					
White meats—at least once a day	**45**	11	17	33	A or D > C or B
Refined groats—at least once a day	**43**	7	12	28	A > D > C or B
Eggs—at least once a day	**47**	6	17	36	A or D > C > B
Red meats—at least once a day	**21**	**15**	12	14	ns
Fried foods—at least once a day	**35**	25	27	18	A > D
Wholemeal groats—at least once a day	**26**	4	5	**26**	A or D > B or C
Processed meats—at least once a day	36	**79**	45	36	B > C > A or D
Refined bread—at least once a day	30	**78**	55	26	B > C > A or D
Butter—at least once a day	45	**80**	50	45	B > C or A or D
Cheese—at least once a day	21	**42**	27	18	B > C or A or D
Sweets—at least once a day	19	**45**	30	22	B > C or A or D; C > A
Tinned meats—at least 1–3 times/week	1	**8**	3	3	B > A
Sweetened drinks—at least once a day	11	22	**29**	7	C > A or D; B > A or D
Energy drinks—at least 1–3 times/week	12	8	**21**	3	C > B or D; A > D
Alcohol—at least 1–3 times/week	23	31	**44**	21	C > B or A or D
Fast foods—at least 1–3 times/week	7	9	**18**	2	C > B or A or D; B > D
Fruit—at least once a day	69	53	33	**78**	D > B > C; A > B > C
Vegetables—at least once a day	74	58	40	**81**	D > B > C; A > B > C
Fermented milk beverages—at least once a day	36	17	21	**43**	D > C or B; A > C or B
Wholemeal bread—at least once a day	35	24	20	**55**	D > A > C
Fish—at least 1–3 times/week	34	12	15	**40**	D > C or B; A > C or B
Cottage cheese—at least once a day	19	12	11	**19**	ns
Milk—at least once a day	44	37	40	**53**	D > C or B
Legumes—at least 1–3 times/week	17	6	8	**25**	D > C or B; A > C or B
Lard—at least 1–3 times/week	8	4	3	7	ns
**Lifestyles behaviours**					
5 or more meals per day	**47**	18	19	**46**	A or D > B or C
High recreational physical activity	**64**	28	41	**59**	A or D > C > B
Current smoking	35	22	**44**	21	C or A > B or D
Smoking in the past	35	45	**64**	53	C > B or A; D >A
High physical activity at work or at school	27	19	26	**31**	D > B
Screen time ≥8 h	16	34	20	16	B > C or A or D

^1^ Components of DLPs are sorted by loading factors (drawn from principal component analysis) from 1st to 4th dietary-lifestyle pattern within dietary components and lifestyle components (see Table S2 in Supplementary Material). Chosen categories of components of DLPs are presented. **Bold font** presents numerical data related to components of DLPs with factor loadings ≥ 0.30 (see Table S2 in Supplementary Material). Statistical significance (Person’s chi-squared test) at *p* < 0.05.

**Table 5:** Adjusted ^1^ associations between dietary-lifestyle patterns (DLPs) and adiposity (*n* = 358): odds ratios (95% Confidence Intervals).

Adherence ^2^ to DLPs	Overweight (BMI = 25–29.9 kg/m^2^)	Central Obesity (WHtR ≥ 0.5)	General Obesity (Body Fat ≥ 25%)	Excess of Visceral Fat Tissue (≥ Me, i.e., 1.565 l)	Increased Skeletal Muscle Mass (≥ Me, i.e., 37%)
	Ref.: 18.5–24.9 kg/m^2^	Ref.: < 0.5	Ref.: < 20%	Ref.: < Me	Ref.: < Me
**Protein food, fried-food and recreational physical activity DLP**					
Lower	1.00	1.00	1.00	1.00	1.00
Moderate	1.02 (0.56; 1.86)	0.54 ** (0.31; 0.95)	0.55 (0.28; 1.09)	0.56 * (0.32; 0.99)	1.53 (0.88;2.66)
Higher	2.22 * (1.19; 4.15)	0.65 (0.37; 1.13)	0.23 **** (0.11; 0.45)	0.45 ** (0.26; 0.79)	2.02 * (1.17; 3.50)
**Sandwiches and convenient diet DLP**					
Lower	1.00	1.00	1.00	1.00	1.00
Moderate	0.71 (0.39; 1.28)	1.18 (0.67; 2.09)	2.27 * (1.12; 4.59)	1.87 * (1.06; 3.31)	0.54 * (0.31;0.94)
Higher	0.68 (0.39; 1.21)	1.99 * (1.14; 3.47)	3.45 **** (1.77; 6.83)	2.59 *** (1.48; 4.54)	0.53 * (0.31; 0.90)
**Fast foods and stimulants DLP**					
Lower	1.00	1.00	1.00	1.00	1.00
Moderate	0.89 (0.51; 1.56)	1.42 (0.81; 2.49)	1.84 (0.92; 3.65)	1.68 (0.95; 2.95)	0.81 (0.48; 1.39)
Higher	0.91 (0.50; 1.65)	2.07 * (1.13; 3.78)	4.76 *** (2.10; 10.74)	3.17 *** (1.68; 5.98)	0.48 * (0.27; 0.86)
**Healthy diet, active at work, past smokers DLP**					
Lower	1.00	1.00	1.00	1.00	1.00
Moderate	1.71 (0.94; 3.10)	1.28 (0.73; 2.25)	0.73 (0.37; 1.48)	0.98 (0.55; 1.76)	0.97 (0.55; 1.69)
Higher	3.35 **** (1.82; 6.18)	0.79 (0.45; 1.39)	0.38 ** (0.19; 0.74)	0.51 * (0.29; 0.89)	1.47 (0.86; 2.51)

^1^ Odds ratios adjusted for age (continuous variable), place of residence (categorical variable), financial situation (categorical variable) and education (categorical variable); ^2^ Adherence to the DLP is based on subjects’ tertile distribution: bottom tertile = lower adherence (used as the reference), middle tertile = moderate adherence, upper tertile = higher adherence; BMI—body mass index; WHtR—waist to height ratio; Me—median; Statistical significance (Wald test): * *p* < 0.05, ** *p* < 0.01, *** *p* < 0.001, **** *p* < 0.0001.

**Table 6:** Adjusted ^1^ associations between dietary-lifestyle patterns (DLPs) and metabolic abnormalities (*n* = 358): odds ratios (95% Confidence Intervals).

Adherence ^2^ to DLPs	Elevated FBG (≥ 100 mg/dL)	Elevated TG (≥ 150 mg/dL)	Elevated TC (≥ 200 mg/dL)	Elevated SBP (≥ 130 mmHg) or DBP (≥ 85 mmHg)	At Least 2 Metabolic Abnormalities
	Ref.: < 100 mg/dL	Ref.: < 150 mg/dL	Ref.: < 200 mg/dL	Ref.: SBP < 130 and DBP < 85	Ref.: No Metabolic Abnormalities
**Protein food, fried-food and recreational physical activity DLP**					
Lower	1.00	1.00	1.00	1.00	1.00
Moderate	0.78 (0.32; 1.91)	0.57 (0.32; 1.01)	0.58 (0.33; 1.02)	0.88 (0.51; 1.52)	0.36 * (0.16; 0.79)
Higher	1.05 (0.43; 2.57)	0.63 (0.35; 1.13)	0.44 ** (0.25; 0.79)	1.15 (0.67; 1.99)	0.49 (0.23; 1.06)
**Sandwiches and convenient diet DLP**					
Lower	1.00	1.00	1.00	1.00	1.00
Moderate	2.15 (0.84; 5.49)	2.07 * (1.13; 3.79)	1.28 (0.70; 2.32)	0.62 (0.37; 1.07)	1.32 (0.62; 2.83)
Higher	1.78 (0.69; 4.64)	1.87 * (1.03; 3.39)	2.72 *** (1.53; 4.86)	0.83 (0.48; 1.41)	2.54 * (1.20;5.39)
**Fast foods and stimulants DLP**					
Lower	1.00	1.00	1.00	1.00	1.00
Moderate	1.11 (0.45; 2.75)	0.90 (0.50; 1.61)	1.40 (0.80; 2.45)	0.86 (0.50; 1.47)	1.02 (0.48; 2.14)
Higher	1.23 (0.50; 3.04)	1.68 (0.92; 3.05)	1.59 (0.88; 2.89)	1.41 (0.82; 2.43)	1.41 (0.66; 3.01)
**Healthy diet, active at work, past smokers DLP**					
Lower	1.00	1.00	1.00	1.00	1.00
Moderate	1.14 (0.51; 2.54)	1.02 (0.57; 1.81)	0.88 (0.50; 1.56)	0.83 (0.49; 1.42)	0.72 (0.34; 1.54)
Higher	0.32 * (0.11; 0.92)	0.99 (0.54; 1.83)	0.76 (0.43; 1.35)	0.90 (0.53; 1.52)	0.64 (0.29; 1.40)

^1^ Odds ratios adjusted for age (continuous variable), place of residence (categorical variable), financial situation (categorical variable) and education (categorical variable); ^2^ Adherence to the DLP is based on subjects’ tertile distribution: bottom tertile = lower adherence (used as the reference), middle tertile = moderate adherence, upper tertile = higher adherence; FBG—fasting blood glucose; TG—triglycerides; TC—total cholesterol; SBP—systolic blood pressure; DBP—diastolic blood pressure; Statistical significance (Wald test): * *p* < 0.05, ** *p* < 0.01, *** *p* < 0.001.

## Supplementary Materials

The following are available online at https://www.mdpi.com/2072-6643/12/3/751/s1, Table S1: Sample characteristic by dietary behaviours and age groups. Table S2: Matrix of factor loadings of PCA-driven dietary-lifestyle patterns identified in the sample (*n* = 358). Table S3: Protein food, fried-food and recreational physical activity dietary-lifestyle pattern (DLP): median (interquartile range) or sample distribution (%) of DLP components by adherence to this DLP (*n* = 358). Table S4: Sandwiches and convenient diet dietary-lifestyle pattern (DLP): median (interquartile range) or sample distribution (%) of DLP components by adherence to this DLP (*n* = 358). Table S5: Fast foods and stimulants dietary-lifestyle pattern (DLP): median (interquartile range) or sample distribution (%) of DLP components by adherence to this DLP (*n* = 358). Table S6: Healthy diet, active, past smokers dietary-lifestyle pattern (DLP): median (interquartile range) or sample distribution (%) of DLP components by adherence to this pattern (*n* = 358). Table S7: Occurrence of adiposity and metabolic abnormalities (%) by adherence to the dietary-lifestyle patterns in the study sample (*n* = 358). Table S8: Crude associations between dietary-lifestyle patterns (DLPs) and adiposity (*n* = 358): odds ratios (95% Confidence Intervals (95% CIs). Table S9: Crude associations between dietary-lifestyle patterns (DLPs) and metabolic abnormalities (*n* = 358): odds ratios (95% Confidence Intervals (95% CI).

## References

[1] Olson J.S., Hummer R.A., Harris K.M. (2017). Gender and Health Behavior Clustering among U.S. Young Adults. Biodemogr. Soc. Biol..

[2] Lawrence E.M., Mollborn S., Hummer R.A. (2017). Health lifestyles across the transition to adulthood: Implications for health. Soc. Sci. Med..

[3] Nutrition Evidence Library (2014). A Series of Systematic Reviews on the Relationship between Dietary Patterns and Health Outcomes.

[4] European Commission (2011). The State of Men’s Health in Europe: Extended Report, Luxembourg, European Commission. http://ec.europa.eu/health/population_groups/docs/men_health_extended_en.pdf.

[5] World Health Organization (WHO) (2013). Global Action Plan for the Prevention and Control of Noncommunicable Diseases 2013–2020. http://apps.who.int/iris/bitstream/10665/94384/1/9789241506236_eng.pdf.

[6] White A., McKee M., de Sousa B., de Visser R., Hogston R., Madsen S.A., Makara P., Richardson N., Zatoński W., Raine G. (2014). An examination of the association between premature mortality and life expectancy among men in Europe. Eur. J. Public Health.

[7] Chella Krishnan K., Mehrabian M., Lusis A.J. (2018). Sex differences in metabolism and cardiometabolic disorders. Curr. Opin. Lipidol..

[8] Bingham C.M.L., Jallinoja P., Lahti-Koski M., Absetz P., Paturi M., Pihlajamäki H., Sahi T., Uutela A. (2010). Quality of diet and food choices of Finnish young men: A sociodemographic and health behaviour approach. Public Health Nutr..

[9] El Ansari W., Stock C., Mikolajczyk R.T. (2012). Relationships between food consumption and living arrangements among university students in four European countries—A cross-sectional study. Nutr. J..

[10] Zadarko-Domaradzka M., Barabasz Z., Sobolewski M., Nizioł-Babiarz E., Penar-Zadarko B., Szybisty A., Zadarko E. (2018). Alcohol Consumption and Risky Drinking Patterns among College Students from Selected Countries of the Carpathian Euroregion. BioMed Res. Int..

[11] European Monitoring Centre for Drugs and Drug Addiction (EMCDDA) (2019). Poland: Country Drug Report. http://www.emcdda.europa.eu/countries/drug-reports/2019/poland/drug-use_en.

[12] Díaz-Gutiérrez J., Ruiz-Canela M., Gea A., Fernández-Montero A., Martínez-González M.Á. (2018). Association between a Healthy Lifestyle Score and the Risk of Cardiovascular Disease in the SUN Cohort. Rev. Esp. Cardiol. (Engl. Ed.).

[13] Hu F.B. (2002). Dietary pattern analysis: A new direction in nutritional epidemiology. Curr. Opin. Lipidol..

[14] Noble N., Paul C., Turon H., Oldmeadow C. (2015). Which modifiable health risk behaviours are related? A systematic review of the clustering of Smoking, Nutrition, Alcohol and Physical activity (‘SNAP’) health risk factors. Prev. Med..

[15] Northstone K., Emmett P. (2010). Dietary patterns of men in ALSPAC: Associations with socio-demographic and lifestyle characteristics, nutrient intake and comparison with women’s dietary patterns. Eur. J. Clin. Nutr..

[16] Greene G.W., Schembre S.M., White A.A., Hoerr S.L., Lohse B., Shoff S., Horacek T., Riebe D., Patterson J., Phillips B.W. (2011). Identifying clusters of college students at elevated health risk based on eating and exercise behaviors and psychosocial determinants of body weight. J. Am. Diet Assoc..

[17] Laska M.N., Pasch K.E., Lust K., Story M., Ehlinger E. (2009). Latent class analysis of lifestyle characteristics and health risk behaviors among college youth. Prev. Sci..

[18] Levinson D.J. (1986). A conception of adult development. Am. Psychol..

[19] Gawecki J. (2018). Dietary Habits and Nutrition Beliefs Questionnaire and the Manual for Developing Nutritional Data.

[20] Kowalkowska J., Wadolowska L., Czarnocinska J., Czlapka-Matyasik M., Galinski G., Jezewska-Zychowicz M., Bronkowska M., Dlugosz A., Loboda D., Wyka J. (2018). Reproducibility of a Questionnaire for Dietary Habits, Lifestyle and Nutrition Knowledge Assessment (KomPAN) in Polish Adolescents and Adults. Nutrients.

[21] ISAK (2001). International Standards for Anthropometric Assessment.

[22] Obesity: Preventing and managing the global epidemic (2000). Report of a WHO consultation. World Health Organ. Technol. Rep. Ser..

[23] World Health Organization (WHO) (2008). Waist Circumference and Waist-Hip Ratio.

[24] Dympna G., Heymsfield S.B., Heo M., Jebb S., Murgatroyd P., Sakamoto Y. (2000). Healthy percentage body fat ranges: An approach for developing guidelines based on body mass index. Am. J. Clin. Nutr..

[25] National Institute for Health and Care Excellence (NICE) Hypertension in Adults: Diagnosis and managment.Clinical guideline NG136. https://www.nice.org.uk/guidance/ng136.

[26] (2001). Executive Summary of the Third Report of the National Cholesterol Education Program (NCEP) Expert Panel on Detection, Evaluation, and Treatment of High Blood Cholesterol in Adults (Adult Treatment Panel III). JAMA.

[27] Alberti K.G., Zimmet P., Shaw J. (2006). Metabolic syndrome—A new world-wide definition. A Consensus Statement from the International Diabetes Federation. Diabet. Med..

[28] Williams B., Mancia G., Spiering W., Rosei E.A., Azizi M., Burnier M., Clement D.L., Coca A., de Simone G., Dominiczak A. (2018). 2018 ESC/ESH Guidelines for the management of arterial hypertension. Eur. Heart J..

[29] Field A. (2009). Discovering Statistics Using SPSS.

[30] Czekajło A., Różańska D., Zatońska K., Szuba A., Regulska-Ilow B. (2019). Association between dietary patterns and metabolic syndrome in the selected population of Polish adults—Results of the PURE Poland Study. Eur. J. Public Health.

[31] Osadnik T., Pawlas N., Lonnie M., Osadnik K., Lejawa M., Wądołowska L., Bujak K., Fronczek M., Reguła R., Gawlita M. (2018). Family History of Premature Coronary Artery Disease (P-CAD)-A Non-Modifiable Risk Factor? Dietary Patterns of Young Healthy Offspring of P-CAD Patients: A Case-Control Study (MAGNETIC Project). Nutrients.

[32] Hu F.B., Rimm E.B., Stampfer M.J., Ascherio A., Spiegelman D., Willett W.C. (2000). Prospective study of major dietary patterns and risk of coronary heart disease in men. Am. J. Clin. Nutr..

[33] Rodríguez-Monforte M., Flores-Mateo G., Sánchez E. (2015). Dietary patterns and CVD: A systematic review and meta-analysis of observational studies. Br. J. Nutr..

[34] Yavari A., Javadi M., Mirmiran P., Bahadoran Z. (2015). Exercise-induced oxidative stress and dietary antioxidants. Asian J. Sports Med..

[35] Ortega F.B., Sui X., Lavie C.J., Blair S.N. (2016). Body Mass Index, the Most Widely Used But Also Widely Criticized Index: Would a Criterion Standard Measure of Total Body Fat Be a Better Predictor of Cardiovascular Disease Mortality?. Mayo Clin. Proc..

[36] Thomas E.L., Frost G., Taylor-Robinson S.D., Bell J.D. (2012). Excess body fat in obese and normal-weight subjects. Nutr. Res. Rev..

[37] Patterson M.L. (1994). Interaction behavior and person perception: An integrative approach. Small Group Res..

[38] Hatcher L. (1994). A Step-By-Step Approach to Using the SAS^®^ System for Factor Analysis and Structural Equation Modeling.

[39] Food and Agriculture Organization (FAO) (2018). Dietary Assessment: A Resource Guide to Method Selection and Application in Low Resource Settings. Rome, Italy..

[40] Wadolowska L. (2005). Walidacja kwestionariusza częstotliwości spożycia żywności–FFQ. Ocena powtarzalności [Validation of food frequency questionnaire–FFQ. Reproducibility assessment]. Bromat. Chem. Toksykol..

[41] Cade J., Thompson R., Burley V., Warm D. (2002). Development, validation and utilization of food-frequency-questionnaires—A review. Public Health Nutr..

[42] Gibney M.J., Margetts B.M., Kearney J.M., Arab L. (2013). Public Health Nutrition.

[43] Gibson R.S. (2005). Principles of Nutritional Assessment.

[44] Thompson F., Subar A., Coulston A.M., Boushey C.J. (2008). Dietary assessment methodology. Nutrition in the Prevention and Treatment of Disease.

[45] Deshmukh-Taskar P.R., O’Neil C.E., Nicklas T.A., Yang S.-J., Liu Y., Gustat J., Berenson G.S. (2009). Dietary patterns associated with metabolic syndrome, sociodemographic and lifestyle factors in young adults: The Bogalusa Heart Study. Public Health Nutr..

[46] Sur G., Floca E., Kudor-Szabadi L., Sur M.L., Sur D., Samasca G. (2014). The relevance of inflammatory markers in metabolic syndrome. Maedica (Buchar.).

[47] Barabási A., Menichetti G., Loscalzo J. (2020). The unmapped chemical complexity of our diet. Nat. Food.

