# Supplementary material for: Dietary-Lifestyle Patterns Associated with Adiposity and Metabolic Abnormalities in Adult Men under 40 Years Old: A Cross-Sectional Study (MeDiSH Project)
Source: Nutrients. 2020 Mar 12;12(3):751. doi: 10.3390/nu12030751 (PMC7146269; doi:10.3390/nu12030751)
Supplement: Supplementary file 1 [file nutrients-12-00751-s001.pdf]

SUPLEMENTARY MATERIAL

Table S1. Sample characteristic by dietary behaviours and age groups.

**Foods [1]**	**Foods frequency consumption (times/day [2])**						*p*-value of Kruskal-Wallis test	*p*-value of Pearson's chi-squared test
	**Total**		**19-30 y**		**31-40 y**			
	**Mean**	**Median (IQR)**	**Mean**	**Median (IQR)**	**Mean**	**Median (IQR)**		
Number of subjects	358		176		182			
Butter	0.87	1.00 (0.14-1.00)	0.80	0.50 (0.14-1.00)	0.93	1.00 (0.14-2.00)	ns	ns
Refined bread	0.76	0.50 (0.14-1.00)	0.69	0.50 (0.14-1.00)	0.82	0.50 (0.14-1.00)	ns	*
Vegetables	0.91	1.00 (0.50-1.00)	0.85	0.50 (0.50-1.00)	0.98	1.00 (0.50-1.00)	**	*
Milk	0.75	0.50 (0.14-1.00)	0.76	0.50 (0.14-1.00)	0.73	0.50 (0.14-1.00)	ns	ns
Fruit	0.84	1.00 (0.50-1.00)	0.74	0.50 (0.50-1.00)	0.94	1.00 (0.50-1.00)	***	**
Processed meats	0.73	0.50 (0.50-1.00)	0.67	0.50 (0.50-1.00)	0.79	0.50 (0.50-1.00)	*	ns
Wholemeal bread	0.58	0.50 (0.14-1.00)	0.49	0.50 (0.14-0.50)	0.66	0.50 (0.14-1.00)	*	*
Refined groats	0.47	0.50 (0.14-0.50)	0.56	0.50 (0.14-0.50)	0.39	0.14 (0.14-0.50)	**	*
Sweets	0.52	0.50 (0.14-1.00)	0.47	0.50 (0.14-0.50)	0.57	0.50 (0.14-1.00)	ns	**
Eggs	0.51	0.50 (0.14-0.50)	0.54	0.50 (0.14-0.50)	0.48	0.50 (0.14-0.50)	ns	ns
Fermented milk beverages	0.46	0.50 (0.06-0.50)	0.46	0.50 (0.06-0.50)	0.46	0.50 (0.06-0.50)	ns	ns
Sweetened drinks	0.29	0.06 (0.06-0.50)	0.34	0.14 (0.06-0.50)	0.25	0.06 (0.06-0.14)	*	ns
White meats	0.55	0.50 (0.50-0.50)	0.59	0.50 (0.50-0.50)	0.51	0.50 (0.50-0.50)	ns	ns
Cheese	0.50	0.50 (0.14-0.50)	0.46	0.50 (0.14-0.50)	0.53	0.50 (0.14-0.50)	ns	ns
Cottage cheese	0.35	0.14 (0.06-0.50)	0.34	0.14 (0.06-0.50)	0.36	0.14 (0.06-0.50)	ns	ns
Fried foods	0.51	0.50 (0.14-0.50)	0.57	0.50 (0.50-0.50)	0.45	0.50 (0.14-0.50)	**	*
Wholemeal groats	0.32	0.14 (0.06-0.50)	0.31	0.14 (0.06-0.50)	0.33	0.14 (0.06-0.50)	ns	*
Red meats	0.36	0.50 (0.06-0.50)	0.38	0.50 (0.10-0.50)	0.35	0.32 (0.06-0.50)	ns	ns
Energy drinks	0.08	0.00 (0.00-0.06)	0.11	0.06 (0.00-0.06)	0.06	0.00 (0.00-0.06)	**	ns
Fish	0.19	0.14 (0.06-0.14)	0.18	0.14 (0.06-0.14)	0.20	0.14 (0.06-0.14)	ns	ns
Alcohol	0.19	0.14 (0.06-0.14)	0.19	0.14 (0.06-0.14)	0.19	0.14 (0.06-0.14)	ns	ns
Fast foods	0.11	0.06 (0.06-0.14)	0.11	0.06 (0.06-0.14)	0.11	0.06 (0.06-0.06)	ns	ns

Lard	0.06	0.00 (0.00-0.06)	0.05	0.00 (0.00-0.06)	0.06	0.00 (0.00-0.06)	ns	ns
Legumes	0.13	0.06 (0.06-0.14)	0.13	0.06 (0.06-0.14)	0.13	0.06 (0.06-0.14)	ns	ns
Tinned meats	0.06	0.06 (0.00-0.06)	0.08	0.06 (0.00-0.06)	0.05	0.06 (0.00-0.06)	ns	ns

[1] Foods sorted in accordance to Table 2 in the main text (i.e. by sample percentages of 'few times a day' category); [2] The answers regarding frequency consumption obtained from the respondent (six to choose from) were transformed to food frequencies (in times/day) as follows: never = 0; 1-3 times a month = 0.06; once a month = 0.14; few times a week = 0.5; once a day = 1.0; few times a day = 2.0. IQR – interquartile range. Kruskal-Wallis test was used to verify differences in medians while Pearson's chi-squared test to verify differences in sample distribution across age groups (although data regarding sample distribution by age groups are not shown in this table); Statistical significance: * $p < 0.05$, ** $p < 0.01$, *** $p < 0.001$; ns—statistically insignificant.

Table S2 Matrix of factor loadings of PCA-driven dietary-lifestyle patterns identified in the sample (n = 358).

**Components**	**Dietary-lifestyle patterns**			
	**Protein food, fried-food and recreational physical activity**	**Sandwiches and convenient diet**	**Fast foods and stimulants**	**Healthy diet, active, past smokers**
**Dietary components**				
White meats	0.70			
Refined groats	0.65			
Eggs	0.57			
Red meats	0.51	0.30		
Fried foods	0.49			
Wholemeal groats	0.37			0.38
Processed meats		0.72		
Refined bread		0.63		
Butter		0.60		
Cheese		0.56		
Sweets		0.39		
Tinned meats		0.33		
Sweetened drinks			0.58	
Energy drinks			0.52	
Alcohol			0.46	
Fast foods			0.39	
Fruit			-0.31	0.57
Vegetables			-0.42	0.54
Fermented milk beverages				0.53
Wholegrain bread				0.48
Fish				0.46
Cottage cheese				0.43
Milk				0.42
Legumes				0.37
**Lifestyle components**				
Number of meals per day	0.37			0.40
Recreational physical activity	0.31			0.30
Current smoking			0.62	
Smoking in the past			0.45	0.37
Physical activity at work or school				0.38
**Variance explained [%]**	12.5	7.8	6.4	5.5

PCA – Principal Component Analysis; Factor loadings of >|0.30| are shown in the table. Sorted by factor loadings from 1st to 4th factor within dietary components and lifestyle components. Total variance explained by four dietary-lifestyle patterns is 32.2%. Factor loadings for "lard" and "screen time" were <|0.30| in all factors, hence the data is not shown.

Table S3. Protein food, fried-food and recreational physical activity dietary-lifestyle pattern (DLP): median [1] (interquartile range) or sample distribution (%) of DLP components by adherence to this DLP (n = 358).

**Dietary behaviours: food frequency consumption (times/day) by adherence to the DLPs**					**Lifestyles behaviours (% sample distribution) by adherence to the DLPs**				
**Foods**	Lower	Moderate	Higher	*p*-value	**Variables**	Lower	Moderate	Higher	*p*-value
Wholemeal bread	0.50 (0.06-0.50)	0.50 (0.14-1.00)	0.50 (0.14-1.00)	*	**Number of meals per day**				****
Wholemeal groats	0.06 (0.06-0.14)	0.14 (0.06-0.50)	0.50 (0.14-1.00)	****	1-2	10.2	3.4	0.0	
Milk	0.50 (0.14-1.00)	0.50 (0.50-1.00)	0.50 (0.14-1.00)	ns	3	52.5	26.5	7.4	
Fermented milk beverages	0.14 (0.06-0.50)	0.50 (0.14-0.50)	0.50 (0.14-1.00)	***	4	32.2	48.7	45.4	
Cottage cheese	0.14 (0.06-0.50)	0.14 (0.06-0.50)	0.50 (0.14-0.50)	****	5 or more	5.1	21.8	47.1	
White meats	0.50 (0.14-0.50)	0.50 (0.50-0.50)	0.50 (0.50-1.00)	****	**Physical activity**				***
Fish	0.06 (0.06-0.14)	0.14 (0.06-0.14)	0.14 (0.14-0.50)	****	**at work or at school** [2]				
Legumes	0.06 (0.06-0.06)	0.06 (0.06-0.14)	0.06 (0.06-0.14)	**	Low	64.4	47.9	38.0	
Fruit	0.50 (0.14-1.00)	0.50 (0.50-1.00)	1.00 (0.50-2.00)	****	Moderate	22.9	37.8	34.7	
Vegetables	0.50 (0.50-1.00)	1.00 (0.50-1.00)	1.00 (0.50-2.00)	****	High	12.7	14.3	27.3	
Eggs	0.14 (0.14-0.50)	0.50 (0.14-0.50)	0.50 (0.50-1.00)	****	**Recreational**				****
Refined bread	1.00 (0.14-2.00)	0.50 (0.14-1.00)	0.50 (0.14-1.00)	**	**physical activity** [3]				
Refined groats	0.14 (0.06-0.50)	0.50 (0.14-1.00)	0.50 (0.50-1.00)	****	Low	29.7	11.8	5.0	
Fast foods	0.06 (0.06-0.14)	0.06 (0.06-0.14)	0.06 (0.06-0.06)	ns	Moderate	55.1	45.4	31.4	
Fried foods	0.50 (0.14-0.50)	0.50 (0.14-0.50)	0.50 (0.50-1.00)	****	High	15.2	42.9	63.6	
Butter	1.00 (0.50-2.00)	0.50 (0.14-1.00)	0.50 (0.14-1.00)	**	**Current smoking**	22.0	15.8	9.9	*
Lard	0.00 (0.00-0.06)	0.00 (0.00-0.06)	0.00 (0.00-0.06)	*	**Smoking in the past**	46.6	33.6	35.5	ns
Cheese	0.50 (0.14-0.50)	0.50 (0.50-0.50)	0.50 (0.14-0.50)	*	**Screen time [hours/day]** [4]				**
Processed meats	0.50 (0.50-1.00)	0.50 (0.50-1.00)	0.50 (0.14-1.00)	ns	<2	7.6	8.4	16.5	
Red meats	0.14 (0.06-0.50)	0.50 (0.14-0.50)	0.50 (0.14-0.50)	****	2 to <4	16.1	17.6	28.1	
Sweets	0.50 (0.14-1.00)	0.50 (0.14-1.00)	0.14 (0.14-0.50)	***	4 to <6	22.9	21.0	28.1	
Tinned meats	0.06 (0.00-0.06)	0.06 (0.00-0.06)	0.06 (0.00-0.06)	ns	6 to <8	16.10	21.0	10.7	
Sweetened drinks	0.06 (0.06-0.50)	0.14 (0.06-0.50)	0.06 (0.06-0.14)	*	8 to <10	20.3	21.8	10.7	
Energy drinks	0.00 (0.00-0.06)	0.06 (0.00-0.06)	0.00 (0.00-0.06)	ns	≥10	17.0	10.1	5.8	
Alcohol	0.14 (0.06-0.14)	0.14 (0.06-0.14)	0.14 (0.06-0.14)	ns					

[1] Medians of food frequency consumption were calculated after transformation of the answers regarding frequency consumption obtained from the respondent (six to choose from) to food frequencies (in times/day) as follows: never = 0; 1-3 times a month = 0.06; once a month = 0.14; few times a week = 0.5; once a day = 1.0; few times a day = 2.0. [2] Physical activity at work or at school was categorised as follows: low – over 70% of time sedentary; moderate – about 50% of time sedentary and 50% active; higher – about 70% of time active or physical labour of high intensity; [3] Recreational physical activity was categorised as follows: low – mostly sedentary, watching TV, reading newspapers/book, light house works, walking for 1-2 hours a week; moderate – walking, cycling, exercise, gardening or other light intensity physical activity for 2-3 hours a week; higher – cycling, running, gardening or other sport activities that require physical activity for more than 3 hours a week; [4] Screen time was assessed using question: 'How many hours a day (on average) do you spend watching TV or using a computer (including work)?'; Kruskal-Wallis test was used to verify differences in medians while Pearson's chi-squared test to verify differences in sample distribution across the levels of adherence to the DLP; Statistical significance: * $p < 0.05$, ** $p < 0.01$, *** $p < 0.001$, **** $p < 0.0001$, ns—statistically insignificant.

Table S4. Sandwiches and convenient diet dietary-lifestyle pattern (DLP): median [1] (interquartile range) or sample distribution (%) of DLP components by adherence to this DLP (n = 358).

**Dietary behaviours: food frequency consumption (times/day) by adherence to the DLPs**					**Lifestyles behaviours (% sample distribution) by adherence to the DLPs**				
**Foods**	Lower	Moderate	Higher	*p*-value	**Variables**	Lower	Moderate	Higher	*p*-value
Wholemeal bread	0.50 (0.14-1.00)	0.50 (0.14-1.00)	0.14 (0.06-0.50)	**	**Number of meals per day**				*
Wholemeal groats	0.50 (0.06-1.00)	0.14 (0.06-0.50)	0.06 (0.06-0.50)	****	1-2	3.4	5.1	5.0	
Milk	0.50 (0.14-1.00)	0.50 (0.14-1.00)	0.50 (0.14-1.00)	ns	3	18.5	30.5	36.4	
Fermented milk beverages	0.50 (0.06-1.00)	0.50 (0.06-0.50)	0.14 (0.06-0.50)	ns	4	44.5	41.5	40.5	
Cottage cheese	0.14 (0.06-0.50)	0.14 (0.14-0.50)	0.14 (0.06-0.50)	ns	5 or more	33.6	22.9	18.2	
White meats	0.50 (0.50-1.00)	0.50 (0.50-0.50)	0.50 (0.50-0.50)	**	**Physical activity**				ns
Fish	0.14 (0.06-0.50)	0.14 (0.06-0.14)	0.14 (0.06-0.14)	**	**at work or at school [2]**				
Legumes	0.06 (0.06-0.14)	0.06 (0.06-0.14)	0.06 (0.06-0.14)	ns	Low	48.7	50.0	51.2	
Fruit	1.00 (0.50-1.00)	0.50 (0.50-1.00)	1.00 (0.50-1.00)	*	Moderate	36.1	29.7	29.8	
Vegetables	1.00 (0.50-2.00)	0.50 (0.50-1.00)	1.00 (0.50-1.00)	*	High	15.1	20.3	19.0	
Eggs	0.50 (0.50-1.00)	0.50 (0.14-0.50)	0.50 (0.14-0.50)	****	**Recreational**				****
Refined bread	0.14 (0.06-0.50)	0.50 (0.14-1.00)	1.00 (1.00-2.00)	****	**physical activity [3]**				
Refined groats	0.50 (0.14-1.00)	0.50 (0.14-0.50)	0.14 (0.14-0.50)	****	Low	2.5	16.9	26.5	
Fast foods	0.06 (0.06-0.06)	0.06 (0.06-0.14)	0.06 (0.06-0.14)	ns	Moderate	37.8	48.3	45.4	
Fried foods	0.50 (0.14-0.50)	0.50 (0.14-0.50)	0.50 (0.50-0.50)	*	High	59.7	34.8	28.1	
Butter	0.14 (0.00-0.50)	1.00 (0.50-1.00)	2.00 (1.00-2.00)	****	**Current smoking**	11.8	13.7	22.3	ns
Lard	0.00 (0.00-0.06)	0.00 (0.00-0.06)	0.00 (0.00-0.06)	**	**Smoking in the past**	37.0	33.1	45.4	ns
Cheese	0.14 (0.14-0.50)	0.50 (0.14-0.50)	0.50 (0.50-1.00)	****	**Screen time [hours/day] [4]**				ns
Processed meats	0.50 (0.14-0.50)	0.50 (0.50-1.00)	1.00 (1.00-2.00)	****	<2	13.4	10.2	9.1	
Red meats	0.14 (0.06-0.50)	0.50 (0.14-0.50)	0.50 (0.14-0.50)	**	2 to <4	21.9	21.2	19.0	
Sweets	0.14 (0.14-0.50)	0.50 (0.14-1.00)	0.50 (0.14-1.00)	****	4 to <6	23.5	27.1	21.5	
Tinned meats	0.00 (0.00-0.06)	0.06 (0.00-0.06)	0.06 (0.00-0.06)	****	6 to <8	13.4	17.8	16.5	
Sweetened drinks	0.06 (0.06-0.14)	0.06 (0.06-0.50)	0.14 (0.06-0.50)	****	8 to <10	17.7	13.6	21.5	
Energy drinks	0.00 (0.00-0.06)	0.03 (0.00-0.06)	0.06 (0.00-0.06)	ns	≥10	10.1	10.2	12.4	
Alcohol	0.14 (0.06-0.14)	0.14 (0.06-0.14)	0.14 (0.06-0.50)	*					

[1] Medians of food frequency consumption were calculated after transformation of the answers regarding frequency consumption obtained from the respondent (six to choose from) to food frequencies (in times/day) as follows: never = 0; 1-3 times a month = 0.06; once a month = 0.14; few times a week = 0.5; once a day = 1.0; few times a day = 2.0. [2] Physical activity at work or at school was categorised as follows: low – over 70% of time sedentary; moderate – about 50% of time sedentary and 50% active; higher – about 70% of time active or physical labour of high intensity; [3] Recreational physical activity was categorised as follows: low – mostly sedentary, watching TV, reading newspapers/book, light house works, walking for 1-2 hours a week; moderate – walking, cycling, exercise, gardening or other light intensity physical activity for 2-3 hours a week; higher – cycling, running, gardening or other sport activities that require physical activity for more than 3 hours a week; [4] Screen time was assessed using question: 'How many hours a day (on average) do you spend watching TV or using a computer (including work)?'; Kruskal-Wallis test was used to verify differences in medians while Pearson's chi-squared test to verify differences in sample distribution across the levels of adherence to the DLP; Statistical significance: * $p < 0.05$, ** $p < 0.01$, *** $p < 0.001$, **** $p < 0.0001$, ns—statistically insignificant.

Table S5. Fast foods and stimulants dietary-lifestyle pattern (DLP): median [1] (interquartile range) or sample distribution (%) of DLP components by adherence to this DLP (n = 358).

**Dietary behaviours: food frequency consumption (times/day) by adherence to the DLPs**					**Lifestyles behaviours (% sample distribution) by adherence to the DLPs**				
**Foods**	Lower	Moderate	Higher	*p*-value	**Variables**	Lower	Moderate	Higher	*p*-value
Wholemeal bread	0.50 (0.50-1.00)	0.50 (0.14-0.50)	0.14 (0.06-0.50)	****	**Number of meals per day**				**
Wholemeal groats	0.50 (0.14-0.50)	0.14 (0.06-0.50)	0.14 (0.06-0.50)	****	1-2	0.8	4.2	8.3	
Milk	0.50 (0.14-1.00)	0.50 (0.14-1.00)	0.50 (0.14-1.00)	ns	3	18.5	33.9	33.1	
Fermented milk beverages	0.50 (0.14-1.00)	0.14 (0.06-0.50)	0.50 (0.06-0.50)	*	4	48.7	38.1	39.7	
Cottage cheese	0.14 (0.06-0.50)	0.14 (0.06-0.50)	0.14 (0.06-0.50)	ns	5 or more	31.9	23.7	19.0	
White meats	0.50 (0.50-0.50)	0.50 (0.14-0.50)	0.50 (0.50-0.50)	ns	**Physical activity**				**
Fish	0.14 (0.06-0.50)	0.14 (0.06-0.14)	0.14 (0.06-0.14)	**	**at work or at school [2]**				
Legumes	0.06 (0.06-0.14)	0.06 (0.06-0.14)	0.06 (0.06-0.14)	ns	Low	53.8	57.6	38.8	
Fruit	1.00 (1.00-2.00)	0.50 (0.50-1.00)	0.50 (0.50-1.00)	****	Moderate	29.4	31.4	34.7	
Vegetables	1.00 (1.00-2.00)	0.50 (0.50-1.00)	0.50 (0.50-1.00)	****	High	16.8	11.0	26.5	
Eggs	0.50 (0.14-1.00)	0.50 (0.14-0.50)	0.50 (0.14-0.50)	*	**Recreational**				ns
Refined bread	0.14 (0.06-1.00)	0.50 (0.14-1.00)	1.00 (0.50-1.00)	****	**physical activity [3]**				
Refined groats	0.50 (0.14-0.50)	0.50 (0.14-0.5)	0.50 (0.14-0.50)	ns	Low	12.6	19.5	14.1	
Fast foods	0.06 (0.06-0.06)	0.06 (0.06-0.14)	0.06 (0.06-0.14)	****	Moderate	38.7	48.3	44.6	
Fried foods	0.50 (0.14-0.50)	0.50 (0.50-0.50)	0.50 (0.50-1.00)	****	High	48.7	32.2	41.3	
Butter	1.00 (0.14-2.00)	1.00 (0.14-1.00)	0.50 (0.14-1.00)	ns	**Current smoking**	0.0	3.4	43.8	****
Lard	0.00 (0.00-0.06)	0.00 (0.00-0.06)	0.00 (0.00-0.06)	ns	**Smoking in the past**	23.5	27.1	64.5	****
Cheese	0.50 (0.14-0.50)	0.50 (0.14-0.50)	0.50 (0.14-1.00)	*	**Screen time [hours/day] [4]**				ns
Processed meats	0.50 (0.50-1.00)	0.50 (0.50-1.00)	0.50 (0.50-1.00)	ns	<2	10.1	11.9	10.7	
Red meats	0.14 (0.06-0.50)	0.14 (0.06-0.50)	0.50 (0.14-0.50)	*	2 to <4	19.3	15.2	27.3	
Sweets	0.50 (0.14-1.00)	0.50 (0.14-0.50)	0.50 (0.14-1.00)	ns	4 to <6	20.2	24.6	27.3	
Tinned meats	0.06 (0.00-0.06)	0.06 (0.00-0.06)	0.06 (0.00-0.06)	ns	6 to <8	14.3	18.6	14.9	
Sweetened drinks	0.06 (0.00-0.14)	0.06 (0.06-0.14)	0.50 (0.06-1.00)	****	8 to <10	20.2	19.5	13.2	
Energy drinks	0.00 (0.00-0.06)	0.00 (0.00-0.06)	0.06 (0.00-0.14)	****	≥10	16.0	10.2	6.6	
Alcohol	0.06 (0.06-0.14)	0.14 (0.06-0.14)	0.14 (0.14-0.50)	****					

[1] Medians of food frequency consumption were calculated after transformation of the answers regarding frequency consumption obtained from the respondent (six to choose from) to food frequencies (in times/day) as follows: never = 0; 1-3 times a month = 0.06; once a month = 0.14; few times a week = 0.5; once a day = 1.0; few times a day = 2.0. [2] Physical activity at work or at school was categorised as follows: low – over 70% of time sedentary; moderate – about 50% of time sedentary and 50% active; higher – about 70% of time active or physical labour of high intensity; [3] Recreational physical activity was categorised as follows: low – mostly sedentary, watching TV, reading newspapers/book, light house works, walking for 1-2 hours a week; moderate – walking, cycling, exercise, gardening or other light intensity physical activity for 2-3 hours a week; higher – cycling, running, gardening or other sport activities that require physical activity for more than 3 hours a week; [4] Screen time was assessed using question: 'How many hours a day (on average) do you spend watching TV or using a computer (including work)?'; Kruskal-Wallis test was used to verify differences in medians while Pearson's chi-squared test to verify differences in sample distribution across the levels of adherence to the DLP; Statistical significance: * $p < 0.05$, ** $p < 0.01$, *** $p < 0.001$, **** $p < 0.0001$, ns—statistically insignificant.

Table S6. Healthy diet, active, past smokers dietary-lifestyle pattern (DLP): median [1] (interquartile range) or sample distribution (%) of DLP components by adherence to this pattern (n = 358).

**Dietary behaviours: food frequency consumption (times/day) by adherence to the DLPs**					**Lifestyles behaviours (% sample distribution) by adherence to the DLPs**				
**Foods**	Lower	Moderate	Higher	*p*-value	**Variables**	Lower	Moderate	Higher	*p*-value
Wholemeal bread	0.14 (0.06-0.50)	0.50 (0.14-0.50)	1.00 (0.50-1.00)	****	**Number of meals per day**				****
Wholemeal groats	0.06 (0.06-0.14)	0.14 (0.06-0.50)	0.50 (0.14-1.00)	****	1-2	10.9	2.5	0.0	
Milk	0.50 (0.06-0.50)	1.00 (0.50-1.00)	1.00 (0.50-2.00)	****	3	44.5	30.5	10.7	
Fermented milk beverages	0.14 (0.06-0.50)	0.50 (0.14-0.50)	0.50 (0.50-1.00)	****	4	35.3	48.3	43.0	
Cottage cheese	0.14 (0.06-0.14)	0.14 (0.14-0.50)	0.50 (0.14-0.50)	****	5 or more	9.2	18.6	46.3	
White meats	0.50 (0.14-0.50)	0.50 (0.50-0.50)	0.50 (0.50-1.00)	****	**Physical activity**				****
Fish	0.06 (0.06-0.14)	0.14 (0.06-0.14)	0.14 (0.14-0.50)	****	**at work or at school** [2]				
Legumes	0.06 (0.06-0.06)	0.06 (0.06-0.14)	0.14 (0.06-0.14)	****	Low	68.1	52.5	29.7	
Fruit	0.50 (0.14-0.50)	1.00 (0.50-1.00)	1.00 (1.00-2.00)	****	Moderate	22.7	33.0	39.7	
Vegetables	0.50 (0.50-0.5)	1.00 (0.50-1.00)	1.00 (1.00-2.00)	****	High	9.2	14.4	30.6	
Eggs	0.14 (0.14-0.50)	0.50 (0.14-0.50)	0.50 (0.50-1.00)	****	**Recreational**				****
Refined bread	1.00 (0.50-2.00)	0.50 (0.14-1.00)	0.50 (0.14-1.00)	***	**physical activity** [3]				
Refined groats	0.14 (0.14-0.50)	0.50 (0.14-0.50)	0.50 (0.14-1.00)	****	Low	29.4	14.4	2.5	
Fast foods	0.06 (0.06-0.14)	0.06 (0.06-0.14)	0.06 (0.06-0.06)	**	Moderate	47.9	45.8	38.0	
Fried foods	0.50 (0.50-0.50)	0.50 (0.14-0.50)	0.50 (0.14-0.50)	ns	High	22.7	39.8	59.5	
Butter	1.00 (0.14-2.00)	1.00 (0.14-2.00)	0.50 (0.14-1.00)	ns	**Current smoking**	6.7	19.5	21.5	**
Lard	0.00 (0.00-0.06)	0.00 (0.00-0.06)	0.00 (0.00-0.06)	ns	**Smoking in the past**	21.8	40.7	52.9	****
Cheese	0.50 (0.14-0.50)	0.50 (0.14-0.50)	0.50 (0.14-0.50)	ns	**Screen time [hours/day]** [4]				***
Processed meats	0.50 (0.50-1.00)	0.50 (0.50-1.00)	0.50 (0.50-1.00)	ns	<2	3.4	9.3	19.8	
Red meats	0.14 (0.06-0.50)	0.14 (0.14-0.50)	0.50 (0.14-0.50)	*	2 to <4	13.4	16.9	31.4	
Sweets	0.50 (0.14-1.00)	0.50 (0.14-1.00)	0.50 (0.14-0.50)	*	4 to <6	24.4	28.8	19.0	
Tinned meats	0.06 (0.00-0.06)	0.06 (0.00-0.06)	0.06 (0.00-0.06)	ns	6 to <8	20.2	14.4	13.2	
Sweetened drinks	0.14 (0.06-0.50)	0.14 (0.06-0.50)	0.06 (0.06-0.14)	***	8 to <10	24.4	16.9	11.6	
Energy drinks	0.06 (0.00-0.06)	0.06 (0.00-0.06)	0.00 (0.00-0.06)	*	≥10	14.3	13.6	5.0	
Alcohol	0.14 (0.06-0.14)	0.14 (0.06-0.14)	0.14 (0.06-0.14)	ns					

[1] Medians of food frequency consumption were calculated after transformation of the answers regarding frequency consumption obtained from the respondent (six to choose from) to food frequencies (in times/day) as follows: never = 0; 1-3 times a month = 0.06; once a month = 0.14; few times a week = 0.5; once a day = 1.0; few times a day = 2.0. [2] Physical activity at work or at school was categorised as follows: low – over 70% of time sedentary; moderate – about 50% of time sedentary and 50% active; higher – about 70% of time active or physical labour of high intensity; [3] Recreational physical activity was categorised as follows: low – mostly sedentary, watching TV, reading newspapers/book, light house works, walking for 1-2 hours a week; moderate – walking, cycling, exercise, gardening or other light intensity physical activity for 2-3 hours a week; higher – cycling, running, gardening or other sport activities that require physical activity for more than 3 hours a week; [4] Screen time was assessed using question: 'How many hours a day (on average) do you spend watching TV or using a computer (including work)?'; Kruskal-Wallis test was used to verify differences in medians while Pearson's chi-squared test to verify differences in sample distribution across the levels of adherence to the DLP; Statistical significance: * $p < 0.05$, ** $p < 0.01$, *** $p < 0.001$, **** $p < 0.0001$, ns—statistically insignificant.

Table S7. Occurence of adiposity and metabolic abnormalieties (%) by adherence to the dietary-lifestyle patterns in the study sample (n = 358).

Adiposity and metabolic outcomes	Protein food, fried-food and recreational physical activity				Sandwiches and convenient diet				Fast foods and stimulants				Healthy diet, active, past smokers			
	Lower	Moderate	Higher	p-value	Lower	Moderate	Higher	p-value	Lower	Moderate	Higher	p-value	Lower	Moderate	Higher	p-value
Number of subjects	118	119	121		119	118	121		119	118	121		119	118	121	
**Adiposity outcomes**																
Overweight (BMI = 25-29.9 kg/m^2)	39.8	41.2	55.4	0.009	51.3	42.4	43.0	0.709	50.4	48.3	38.8	0.062	31.9	44.9	59.5	0.002
Central obesity (WHtR ≥ 0.5)	52.2	33.6	35.5	0.005	33.6	36.4	51.2	0.011	37.0	41.5	43.0	0.615	39.5	45.8	36.4	0.322
General obesity (Body fat ≥ 25%)	50.0	29.4	18.2	<0.001	21.8	31.4	43.8	0.001	26.9	31.4	38.8	0.339	37.8	35.6	24.0	0.139
Excess of visceral fat tissue (≥ Me, i.e. 1.565 l)	64.4	46.2	39.7	<0.001	37.8	50.0	62.0	0.001	43.7	50.8	55.4	0.190	53.8	54.2	42.1	0.105
Increased skeletal muscle mass (≥ Me; i.e. 37.0%)	38.1	52.1	59.5	0.004	59.7	47.5	43.0	0.028	52.9	50.8	46.3	0.573	47.9	46.6	55.4	0.341
**Metabolic outcomes**																
Elevated FBG (≥ 100 mg/dL)	12.7	9.2	9.9	0.655	6.7	13.6	11.6	0.213	10.1	10.2	11.6	0.916	11.8	14.4	5.8	0.085
Elevated TG (≥ 150 mg/dL)	38.1	25.2	25.6	0.046	21.0	33.9	33.9	0.042	28.6	26.3	33.9	0.416	28.6	29.7	30.6	0.944
Elevated TC (≥ 200 mg/dL)	45.8	31.9	24.8	0.002	25.2	28.8	47.9	<0.001	31.1	36.4	34.7	0.675	36.1	33.9	32.2	0.815
Elevated SBP (≥130 mmHg) or DBP (≥ 85 mmHg)	42.4	36.1	41.3	0.575	44.5	33.9	41.3	0.230	39.5	34.7	45.4	0.238	42.0	38.1	39.7	0.828
At least 2 metabolic abnormalities	44.1	22.7	25.6	0.001	23.5	27.1	41.3	0.007	30.2	28.0	33.9	0.606	32.8	31.4	28.1	0.723

BMI – body mass index; WHtR – waist to height ratio; Me – median; FBG – fasting blood glucose; TG – triglycerides; TC – total cholesterol; SBP – systolic blood pressure; DBP – diastolic blood pressure. Pearson's chi-squared test was used to verify differences in sample distribution across the levels of adherence to DLP.

Table S8. Crude associations between dietary-lifestyle patterns (DLPs) and adiposity (n = 358): odds ratios (95% Confidence Intervals (95%CIs).

**Adherence [#] to DLPs**	**Overweight (BMI = 25-29.9 kg/m^2)**	**Central obesity (WHtR ≥ 0.5)**	**General obesity (Body fat ≥ 25%)**	**Excess of visceral fat tissue (≥ Me, i.e. 1.565 l)**	**Increased skeletal muscle mass (≥ Me, i.e. 37%)**
	Ref.: 18.5-24.9 kg/m^2	Ref.: < 0.5	Ref.: < 20%	Ref.: < Me	Ref.: < Me
**Protein food, fried-food and recreational physical activity DLP**					
Lower	1.00	1.00	1.00	1.00	1.00
Moderate	0.82	0.46**	0.44**	0.47**	1.76*
	(0.47; 1.43)	(0.27; 0.77)	(0.24; 0.82)	(0.28; 0.80)	(1.05; 2.97)
Higher	1.55	0.50**	0.19****	0.36***	2.38**
	(1.89; 2.71)	(0.30; 0.84)	(0.09; 0.36)	(0.21; 0.61)	(1.41; 4.02)
**Sandwiches and convenient diet DLP**					
Lower	1.00	1.00	1.00	1.00	1.00
Moderate	0.76	1.13	2.02*	1.64	0.61
	(0.44; 1.30)	(0.66; 1.94)	(1.06; 3.83)	(0.98; 2.77)	(0.34; 1.02)
Higher	0.77	2.07**	3.66****	2.68***	0.51*
	(0.45; 1.33)	(1.23; 3.50)	(1.94; 6.89)	(1.58; 4.53)	(0.30; 0.85)
**Fast foods and stimulants DLP**					
Lower	1.00	1.00	1.00	1.00	1.00
Moderate	0.88	1.21	1.31	1.33	0.92
	(0.51; 1.51)	(0.72; 2.04)	(0.70; 2.44)	(0.78; 2.22)	(0.55; 1.54)
Higher	0.69	1.28	1.91*	1.60	0.77
	(0.40; 1.20)	(0.76; 2.16)	(1.03; 3.53)	(0.96; 2.67)	(0.45; 1.28)
**Healthy diet, active at work, past smokers DLP**					
Lower	1.00	1.00	1.00	1.00	1.00
Moderate	1.57	1.23	0.83	1.02	0.95
	(0.90; 2.74)	(0.77; 2.17)	(0.45; 1.52)	(0.61; 1.71)	(0.57; 1.58)
Higher	3.26****	0.87	0.46*	0.63	1.35
	(1.84; 5.78)	(0.52; 1.48)	(0.24; 0.85)	(0.37; 1.04)	(0.81; 2.25)

[1] Adherence to the DLP is based on subjects' tertile distribution: bottom tertile = lower adherence (used as the reference), middle tertile = moderate adherence, upper tertile = higher adherence; BMI – body mass index; WHtR – waist to height ratio; Me – median; Statistical significance (Wald test): * p < 0.05, ** p < 0.01, *** p < 0.001, **** p < 0.0001.

Table S9. Crude associations between dietary-lifestyle patterns (DLPs) and metabolic abnormalities (n = 358): odds ratios (95% Confidence Intervals (95%CI).

**Adherence** [1] **to DLPs**	**Elevated FBG** (≥ 100 mg/dL)	**Elevated TG** (≥ 150 mg/dL)	**Elevated TC** (≥ 200 mg/dL)	**Elevated SBP** (≥ 130 mmHg) or **DBP** (≥ 85 mmHg)	**At least 2 metabolic abnormalities**
	Ref.: < 100 mg/dL	Ref.: < 150 mg/dL	Ref.: < 200 mg/dL	Ref.: SBP < 130 and DBP < 85	Ref.: no metabolic abnormalities
**Protein food, fried-food and recreational physical activity DLP**					
Lower	1.00	1.00	1.00	1.00	1.00
Moderate	0.70	0.55*	0.56*	0.77	0.35**
	(0.31;1.60)	(0.31;0.96)	(0.33;0.95)	(0.46;1.30)	(0.17;0.70)
Higher	0.76	0.56*	0.39***	0.96	0.36**
	(0.34;1.70)	(0.32;0.97)	(0.22;0.68)	(0.57;1.60)	(0.18;0.71)
**Sandwiches and convenient diet DLP**					
Lower	1.00	1.00	1.00	1.00	1.00
Moderate	2.18	1.93*	1.20	0.64	1.38
	(0.89;5.33)	(1.07;3.46)	(0.67;2.14)	(0.37;1.08)	(0.69;2.74)
Higher	1.81	1.93*	2.73***	0.88	2.93**
	(0.73;4.52)	(1.08;3.45)	(1.58;4.73)	(0.52;1.47)	(1.48;5.81)
**Fast foods and stimulants DLP**					
Lower	1.00	1.00	1.00	1.00	1.00
Moderate	1.01	0.89	1.27	0.81	0.89
	(0.38;2.71)	(0.50;1.58)	(0.74;2.18)	(0.48;1.39)	(0.45;1.75)
Higher	1.17	1.28	1.18	1.28	1.14
	(0.51;2.65)	(0.74;2.22)	(0.68;2.02)	(0.76;2.14)	(0.59;2.21)
**Healthy diet, active, past smokers DLP**					
Lower	1.00	1.00	1.00	1.00	1.00
Moderate	1.26	1.05	0.91	0.85	0.81
	(0.59;2.71)	(0.60;1.85)	(0.53;1.55)	(0.50;1.43)	(0.42;1.59)
Higher	0.46	1.10	0.84	0.91	0.75
	(0.18;1.19)	(0.63;1.92)	(0.49;1.44)	(0.54;1.52)	(0.38;1.47)

[1] Adherence to the DLP is based on subjects' tertile distribution: bottom tertile = lower adherence (used as the reference), middle tertile = moderate adherence, upper tertile = higher adherence; FBG – fasting glucose; TG – triglycerides; TC – total cholesterol; SBP – systolic blood pressure; DBP – diastolic blood pressure; Statistical significance (Wald test): * $p < 0.05$, ** $p < 0.01$, *** $p < 0.001$.
